# Glycine N-methyltransferase inhibits aristolochic acid nephropathy by increasing CYP3A44 and decreasing NQO1 expression in female mouse hepatocytes

**DOI:** 10.1038/s41598-018-22298-6

**Published:** 2018-05-03

**Authors:** Ming-Min Chang, Chang-Ni Lin, Cheng-Chieh Fang, Marcelo Chen, Peir-In Liang, Wei-Ming Li, Bi-Wen Yeh, Hung-Chi Cheng, Bu-Miin Huang, Wen-Jeng Wu, Yi-Ming Arthur Chen

**Affiliations:** 1 Center for Infectious Disease and Cancer Research (CICAR), Kaohsiung Medical University, Kaohsiung, Taiwan; 2 Department of Urology, Mackay Memorial Hospital, Taipei, Taiwan; 3 Department of Cosmetic Applications and Management, Mackay Junior College of Medicine, Nursing and Management, Taipei, Taiwan; 4 Department of Pathology, Kaohsiung Medical University Hospital, Kaohsiung Medical University, Kaohsiung, Taiwan; 5 Pingtung Hospital, Ministry of Health and Welfare, Executive Yuan, Pingtung, Taiwan; 6 Department of Urology, School of Medicine, Kaohsiung Medical University, Kaohsiung, Taiwan; 7 Department of Urology, Kaohsiung Medical University Hospital, Kaohsiung, Taiwan; 8 Department of Biochemistry and Molecular Biology, College of Medicine, National Cheng Kung University, Tainan, Taiwan; 9 Department of Urology, Kaohsiung Municipal Ta-Tung Hospital, Kaohsiung, Taiwan; 10 Graduate Institute of Medicine, College of Medicine, Kaohsiung Medical University, Kaohsiung, Taiwan; 11 Present Address: Department of Cell Biology and Anatomy, College of Medicine, National Cheng Kung University, Tainan, Taiwan

## Abstract

Plants containing aristolochic acids (AA) are nephrotoxins. Glycine N-methyltransferase (GNMT) acts to bind environmental toxins such as benzo(a)pyrene and aflatoxin B1, translocate into nucleus, and alter hepatic metabolism. This study aims to determine the role of GNMT in AA-induced nephropathy. We established an AA nephropathy mouse model and found that AA type I (AAI)-induced nephropathy at a lower concentration in male than in female mice, implying sex differences in AAI resistance. Microarray analysis and AAI-treated mouse models showed that GNMT moderately reduced AAI-induced nephropathy by lowering the upregulated level of NQO1 in male, but significantly improved the nephropathy additionally by increasing Cyp3A44/3A41 in female. The protective effects of GNMT were absent in female GNMT knockout mice, in which re-expression of hepatic GNMT significantly decreased AAI-induced nephropathy. Mechanism-wise, AAI enhanced GNMT nuclear translocation, resulting in GNMT interaction with the promoter region of the genes encoding Nrf2 and CAR/PXR, the transcription factors for *NQO1* and *CYP3A44/3A41*, respectively. Unlike the preference for *Nrf2/NQO1* transcriptions at lower levels of GNMT, overexpression of GNMT preferred *CAR/PXR/CYP3A44/3A41* transcriptions and alleviated kidney injury upon AAI treatment. In summary, hepatic GNMT protected mice from AAI nephropathy by enhancing *CAR/PXR/CYP3A44/3A41* transcriptions and reducing *Nrf2/NQO1* transcriptions.

## Introduction

The aristolochic acids (AA) found in *Arostilochia* plant species are classified as Group 1 carcinogens by the World Health Organization (WHO)^1^. Exposure to AA causes aristolochic acid nephropathy (AAN) and Balkan-endemic nephropathy (BEN), which are characterized by progressive renal interstitial fibrosis and tubular atrophy, may slowly progress to end stage renal disease (ESRD), and are frequently associated with urothelial malignancies^2–6^. While AAN occurs worldwide, its incidence is high in Asia and the Balkans. Asian countries, where traditional herbal medicines are widely used, have a high risk for AAN because of the misuse of AA-containing herbs. In the Balkan regions, consumption of AA-contaminated wheat flour is thought to be responsible for the high incidence of BEN^3,6,7^. The botanicals known or suspected of containing AA have been banned and removed from pharmacopeia. However, many illegal products containing AA are still sold via broadcasting radio stations and the internet as health supplements for weight loss, anti-inflammation, rheumatism and pain relief ^8,9^.

The AA family of compounds includes aristolochic acid type I (AAI; C_17_H_11_NO_7_) and its demethoxylated derivative, AA type II (AAII; C_16_H_9_NO_6_). The nephrotoxicity of AAI is much higher than that of AAII^10,11^. AAI is primarily metabolized via two pathways^12–14^. One pathway involves the demethylation of AAI to 8-hydroxyaristolochic acid I (AAIa; C_16_H_9_NO_7_) under aerobic conditions. Carried out by hepatic microsomal cytochromes P450s (e.g. CYP1A, 2C and 3A) in human and rodents, this step is believed to be a detoxification reaction because AAIa has much less renal toxicity and is more readily excreted in urine than AAI^13–16^. Alternatively, in the cytosol of liver and kidney cells, the nitro group of AAI can be enzymatically reduced by nitroreductase, NAD(P)H: quinone oxidoreductase (e.g. NQO1), to generate aristolactam I (ALI; C_17_H_11_NO_4_)^17,18^. The nitroreduction intermediate with a cyclic nitrenium ion interacts with the exocyclic amino groups of deoxyadenosine and deoxyguanosine residues in DNA to form DNA adducts (dA-AAI and dG-AAI)^19–21^. These AAI-DNA adducts have been reported to cause a gene transversion (A:T → T:A), a mutation signature of AA exposure, in upper tract urothelial carcinoma (UTUC)^6,22^ and liver cancer^23^. Inhibition of NQO1 activity suppresses AAI nitroreduction and attenuates its nephrotoxicity, genotoxic and carcinogenic potential^17,18^. However, the pathway of AAI metabolism through which this attenuation occurs is still unknown.

Glycine N-methyltransferase (GNMT) is a multifunctional and tissue-specific protein and abundantly expressed in the liver, pancreas, kidney and prostate^24,25^. This enzyme transfers a methyl group from S-adenosylmethionine (SAM) to glycine to produce S-adenosylhomocysteine (SAH) and sarcosine, a reaction regulated by the binding of 5-methyltetrahydrofolate. GNMT regulates the availability of activated methyl donor SAM for more than a hundred of essential cellular methyltransferase reactions^26–28^. Low GNMT expression has been observed in human hepatoma tissues and liver cancer cell lines^29,30^. Besides, GNMT knockout mice develop chronic hepatitis, glycogen storage disease, steatohepatitis, fibrosis and spontaneous hepatocellular carcinoma (HCC), indicating that GNMT plays an important role in liver function and is a tumor suppressor gene for liver cancer^31–33^. Additionally, GNMT was shown to participate in the cellular defense against environmental toxins such as benzo(a)pyrene (BaP) and aflatoxin B1 (AFB1) by physically binding these xenobiotics^34–38^. Although there is no nuclear localization sequence or classical DNA-binding domain found in GNMT, nuclear translocation of GNMT was induced after BaP and AFB1 exposure^36–38^. Previous studies have suggested that GNMT may participate in and serve as a cofactor for the regulation of detoxification gene expression, such as CYP 1A1 and CYP1A2, thereby decreasing the formations of BaP- and AFB1-DNA adducts^35–39^. However, the function of nuclear GNMT is still unknown.

Here, we aim to delineate the role of GNMT in AAI-induced nephropathy and clarify the molecular mechanism underlying its action. In our *Gnmt* genetically-modified mouse models, we were able to induce AA nephropathy in a C57BL/6 background which was similar to the human histopathology^40,41^ and demostrated that GNMT protected mice from AAI-induced kidney injury by increasing *CAR/PXR* and *CYP3A44/3A41* transcriptions and decreasing *NRF2/NQO1* transcriptions in female mouse hepatocytes.

## Results

### Female mice are more tolerant than male mice to AAI exposure

**Figure 1:**
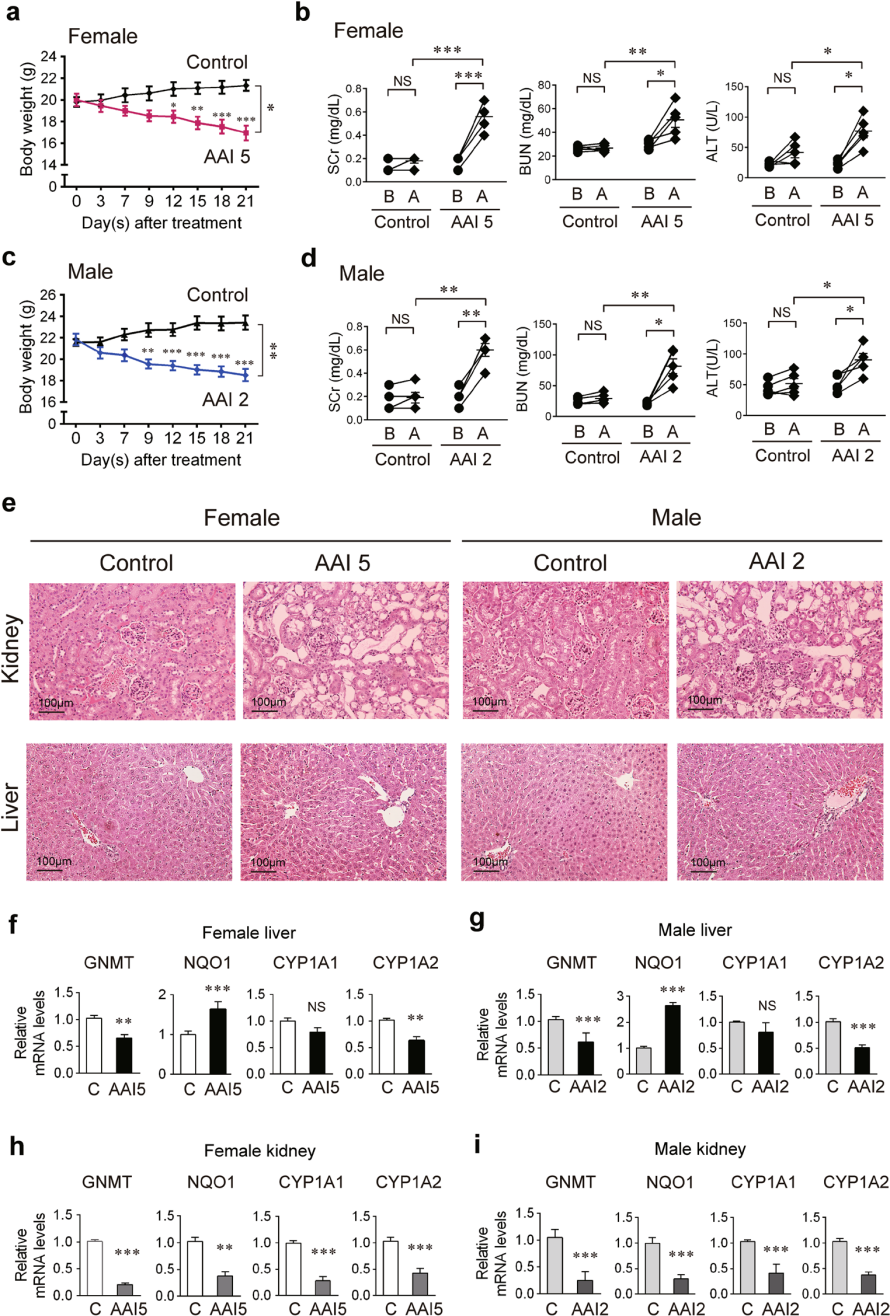
GNMT participates in the metabolism of AAI. (**a,c**) Body weight (BW) of wild-type (WT) C57BL/6 mice intraperitoneally injected with 2 or 5 mg/kg BW/day AAI (AAI 2 or AAI 5) or corn oil (vehicle control), 5 days/week for 3 weeks. Values presented are mean ± SEM, n = 5. *p*-values were calculated by two-way ANOVA and Sidak’s multiple comparisons test. (**b**,**d**) The serum levels of alanine aminotransferase (ALT), creatinine (SCr) and blood urea nitrogen (BUN) were measured from mice at the day before (B) and after (A) the 3-week AAI treatment. The biochemistry parameters from the same mouse before and after AAI-treatment were analyzed by Paired *t*-test. *p*-values for the comparison of biochemistry parameters from AAI- and corn oil-treated mice were calculated by Student’s *t*-test. (**e**) Histological examination of the kidney and liver tissue in AAI- and corn oil-treated WT mice (hematoxylin and eosin stain, original magnification ×200). (**f–i**) mRNA levels of *Gnmt, Nqo1, Cyp1A1, Cyp1A2* genes in the livers (**f**, female liver; **g**, male liver) and kidneys (**h**, female kidney; **i**, male kidney) from AAI- or corn oil-treated WT mice were determined by qPCR. Data were normalized to β-actin. All values are presented as the mean ± SEM, n = 5. *p*-values were calculated by Student’s *t*-test. *p < 0.05, **p < 0.001, ***p ≤ 0.0001.

Sex differences have been extensively studied in patients with chronic kidney disease (CKD), urothelial cancer, and renal cell carcinoma (RCC)^42,43^. Men are at higher risk than women for ESRD and dialysis^44,45^. Men also tend to have worse CKD progression than women^46,47^. In this study, both female and male C57BL/6 wild-type (WT) mice were treated with AAI (5 mg/kg/day) by intraperitoneal (i.p.) injection 5 days a week for 3 weeks^40,41^. The vehicle (corn oil)-treated group served as the normal control. Surprisingly, the majority of AAI-treated male mice (3/5) died on day 5, whereas all the AAI-treated female mice looked well. We presumed that the male mice could not tolerate AAI at the dose of 5 mg/kg/day. Thus, the dose of AAI for male mice was reduced to 2 mg/kg/day. After 3 weeks of treatment, the body weight of female mice significantly decreased in the AAI treatment groups (16.94 ± 0.67 g) as compared with the vehicle-treated group (21.32 ± 0.58 g) by 21% (*P* < 0.0001) (Fig. 1a). The serum biochemistry tests showed that kidney function was impaired after AAI exposure (Fig. 1b). The alanine aminotransferase (ALT) levels were increased after AAI-treatment, but they were not significantly different from that of the control group (Fig. 1b). Similarly, a 22% weight loss and impaired kidney function were noted in the 2 mg/kg/day AAI-treated male mice (Fig. 1c,d). In both sexes, the kidneys from AAI-treated mice were paler in color than those of the control group (Supplementary Fig. S1a), but the kidney weights were not significantly different between the groups (Supplementary Fig. S1b). Furthermore, no differences were observed in the color and size of livers from each group (Supplementary Fig. S1c,d). Histological analysis showed that even though the dose of AAI was 2.5-fold lower for male mice, tubular and interstitial atrophy were clearly widespread in the kidneys of AAI-treated mice of both sexes (Fig. 1e). The livers of both male and female mice were not affected (Fig. 1e). This result suggests that it was renal function that was more tolerant in females, since the impact on liver function were not different between sexes.

### AAI reduces the expression of GNMT

CYP1A2, CYP1A1 and NQO1 have been reported to be the major enzymes involved in AAI metabolism^13,15,48^. To examine whether GNMT participates in AAI-induced nephropathy, the levels of mRNA expression for GNMT, CYP1A1, CYP1A2, and NQO1 in mouse livers and kidneys were determined using real-time quantitative PCR (qPCR) analysis. In both sexes, the liver GNMT and CYP1A2 mRNA levels decreased and liver NQO1 mRNA levels increased significantly after 3 weeks of AAI exposure (Fig. 1f,g). The change in liver CYP1A1 mRNA levels of AAI-treated mice was not significant. In female and male mouse kidneys, the mRNA levels of all these genes decreased significantly after the 3-week AAI exposure, although CYP1A1 and CYP1A2 mRNA levels in kidney were very low and hardly detectable (Fig. 1h,i). To investigate the interaction of AAI and GNMT expression, a luciferase reporter assay was performed by using GNMT promoter-luciferase reporter transfected Huh7 cells (H7GPL cells). The luciferase intensities of AAI-treated H7GPL cells were not significantly different from that of vehicle-treated group. These results indicated that AAI does not directly interact with the GNMT promoter (Supplementary Fig. S2). Furthermore, Li *et al*. reported that miR-224 expression in the kidney is increased in AAI-treated rats^49^. Our unpublished data indicated that miR-224 directly targets the coding sequence (CDS) of GNMT mRNA. Therefore, we investigated the miR-224 expression in the liver and kidneys of AAI-treated mice and found that miR-224 mRNA was increased in the liver and kidneys after 3-week AAI exposure (Supplementary Fig. S3). These results showed that AAI may decrease GNMT expression by increasing miR-224.

### GNMT prevents increase in NQO1 expression in response to AAI

**Figure 2:**
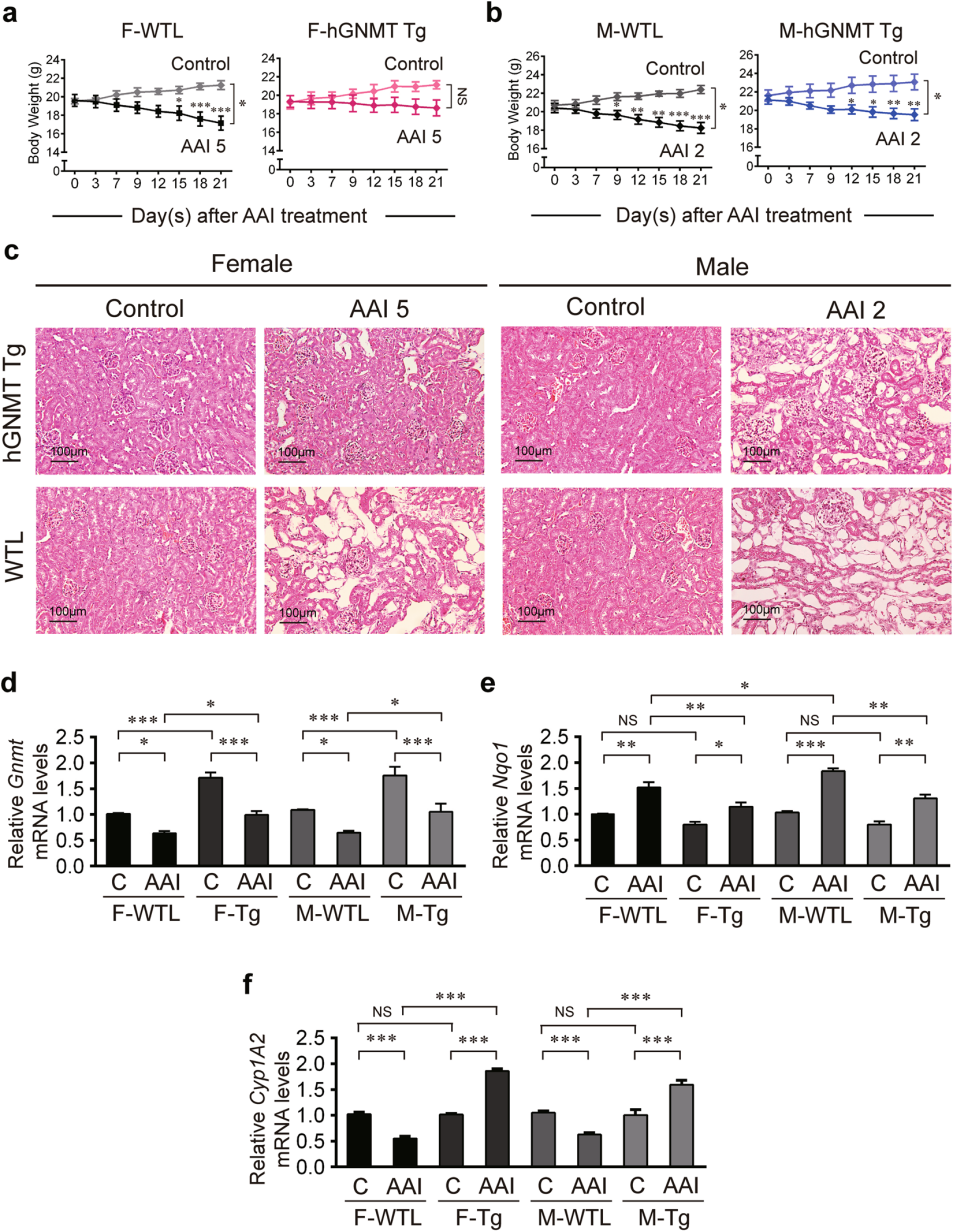
GNMT plays a protective role in AAI-induced renal damage. (**a,b**) Body weight of hGNMT transgenic (hGNMT Tg) mice and their wild-type littermates (WTL) intraperitoneally injected with 2 or 5 mg/kg BW/day AAI (AAI 2 or AAI 5) or corn oil (vehicle control), 5 days a week for 3 weeks. F: female; M: male. Values are presented as the mean ± SEM, n = 4. *p*-values were calculated by two-way ANOVA and Sidak’s multiple comparisons test. (**c**) Histological examination of the kidney morphology after AAI administration in GNMT Tg and WTL mice (hematoxylin and eosin stain, original magnification ×200). (**d–f**) mRNA level of *Gnmt, Nqo1* and *Cyp1A2* genes in the liver of AAI- or corn oil-treated GNMT Tg and WTL mice were determined by qPCR. Data were normalized to β-actin. C: vehicle control. Values are presented as the mean ± SEM, n = 5. *p*-values were calculated using Student’s *t*-test. *p < 0.05, **p < 0.001, ***p ≤ 0.0001.

To confirm whether GNMT is involved in the regulation of AAI metabolism, we used human GNMT transgenic (Tg) mice that overexpress human GNMT in the liver and kidneys^38^. After 3 weeks of AAI i.p. injection, the weight of AAI-treated Tg mice gradually decreased compared to their age- and sex-matched wild-type littermates (WTL) (Fig. 2a,b). Serum creatinine (SCr) in the AAI-treated Tg mice were not elevated as high as that in AAI-treated WTL mice (Supplementary Fig. S4a,b). Serum ALT levels showed that livers of both female and male Tg mice were not affected (Supplementary Fig. S4c,d). Microscopic examination showed that AAI-induced kidney injury (tubular atrophy) was drastically reduced in female Tg mice, but only reduced 30–40% in male Tg mice (Fig. 2c). After 3 weeks of AAI exposure, the GNMT mRNA levels in AAI-treated Tg mice decreased to that of the vehicle-treated WTL mice (Fig. 2d). The fold change in liver NQO1 mRNA in AAI-treated Tg mice was not as high as that in AAI-treated WTL mice (Fig. 2e). The fold change in CYP1A2 mRNA levels increased in Tg mice but decreased in AAI-treated WTL mice (Fig. 2f). However, the CYP1A2 mRNA levels were elevated by AAI treatment in both female and male Tg mice (Fig. 2f), the severe renal damage occurred only in AAI-treated male Tg mice (Fig. 2c). The kidney GNMT, NQO1 and CYP1A2 mRNA levels were decreased in all groups after AAI treatment (Supplementary Fig. S4e–g). These results suggest that overexpression of GNMT exerts protective effects against nephrotoxicity by suppressing the AAI-induced upregulation of NQO1.

### GNMT decreases AAI-induced renal toxicity by increasing the expressions of CYP3A44/CYP3A41 genes

**Figure 3:**
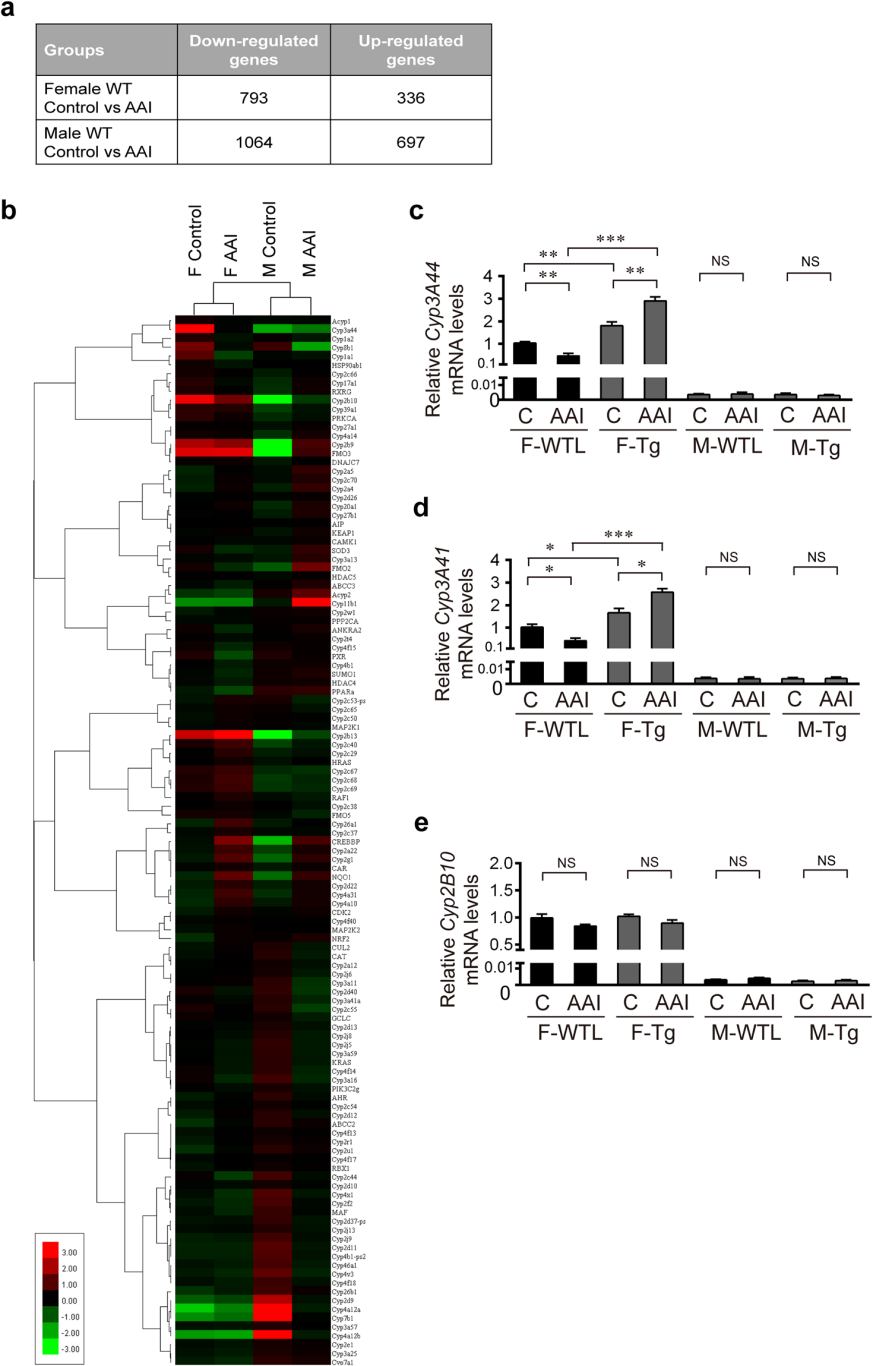
GNMT prevented AAI-induced kidney injury by regulating cytochrome p450 enzymes in female mouse hepatocytes. RNA microarray analysis of the livers from AAI- or corn oil (vehicle control)-treated WT mice, as described in Fig. 1a and c. (**a**) The numbers of signature genes in liver in response to AAI treatment. Genes were identified as significantly changed if the fold change was greater than 2 (up or down) and the *p*-value was less than 0.01 in comparison to the control group. (**b**) Heat map from hierarchical clustering of xenobiotic metabolism genes. Red and green colors indicate an upregulation and downregulation of gene expression, respectively. Trees on the left of the heat map shows the gene clusters. (**c–e**) mRNA level of *Cyp3A44, Cyp3A41*and *Cyp2B10* genes in the liver of AAI- or corn oil-treated hGNMT Tg (Tg) and WTL mice, as described in Fig. 2a and b, were determined by qPCR. Data were normalized to β-actin. (C: vehicle control, F: female, M: male). Values are presented as the mean ± SEM, n = 5. *p*-values were calculated using Student’s *t*-test. *p < 0.05, **p < 0.001, ***p ≤ 0.0001.

We next performed RNA microarray analysis of AAI-treated WT mice, 793 and 336 genes were more than 2-fold down- and up-regulated, respectively, in 5 mg//kg/day AAI-treated female mouse liver, whereas 1064 and 697 genes were more than 2-fold down- and up-regulated in 2 mg/kg/day AAI male mouse liver (Fig. 3a). To screen for hepatic xenobiotic metabolic candidates involved in sex differences and in GNMT regulation, microarray clustering analysis was performed and showed that in female mice, CYP3A44 was highly related to CYP1A2 and CYP1A1 after AAI exposure (Fig. 3b). CYP3A44, CYP2B10, CYP1A2 and CYP1A1 were the most downregulated cytochrome P450 genes in AAI-treated WT female mice.

In mouse, CYP3A44, CYP3A41 and CYP2B10 are female-specific genes^50^. Mouse CYP3A44 and CYP3A41 are the homologues of human CYP3A4, an important enzyme for AAI metabolism^13^. Higher CYP3A4 expression and activity in hepatocytes have been observed in women than in men^51,52^. In human GNMT Tg animal models, CYP3A44 and CYP3A41 but not CYP2B10 were upregulated in female Tg mice (Fig. 3c–e). CYP3A44, CYP3A41 were upregulated in female Tg mice and downregulated in female WTL mice after AAI exposure; however, these genes were not expressed in male mice (Fig. 3c–e)^50^. These results may explain the sex difference in AAI tolerance (Fig. 1a–e) and suggest that GNMT reduced AAI nephrotoxicity by regulating female-specific CYP3A44 and CYP3A41.

### The protective effect of GNMT is lost in GNMT knockout mice

**Figure 4:**
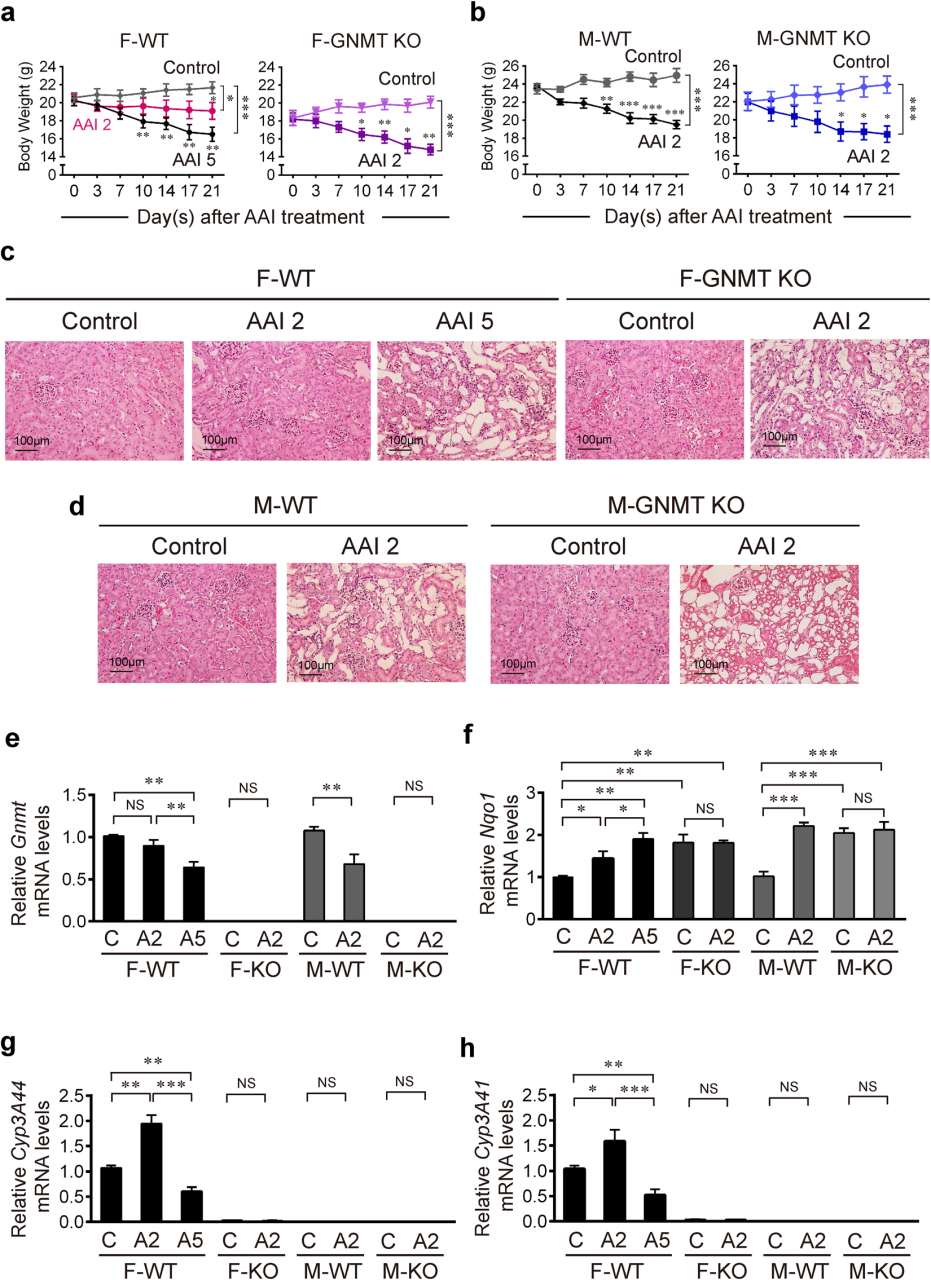
GNMT knockout female mice lose tolerance to AAI. (**a,b**) Body weight of GNMT knockout (GNMT KO) or wild-type (WT) mice intraperitoneally injected with 2 or 5 mg/kg/day AAI (AAI 2 or AAI 5) or corn oil (vehicle control), 5 days/week for 3 weeks. Values are presented as the mean ± SEM, n = 4. *p*-values were calculated by two-way ANOVA and Sidak’s multiple comparisons test. (**c,d**) Histological analysis of renal morphology in corn oil- or AAI-treated mice, as described in (**a**,**b**) (hematoxylin and eosin stain, original magnification ×200). (**e–h**) mRNA level of *Gnmt, Nqo1, Cyp3A4, and Cyp3A41* genes in the liver of AAI- or corn oil-treated WT or GNMT KO mice, as described in (**a**,**b**), were determined by qPCR. Data were normalized to β-actin. (A2, A5: 2 or 5 mg/kg BW/day AAI administration, C: vehicle control) Values are presented as the mean ± SEM, n = 5. *p*-values were calculated using Student’s *t*-test. *p < 0.05, **p < 0.001, ***p ≤ 0.0001.

We then used a GNMT knockout (KO) mouse model to confirm that GNMT protects against AAI nephrotoxicity. After 3 weeks of AAI treatment, the weight loss was similar between GNMT KO female mice treated with low dose AAI (2 mg/kg/day) and WT female mice treated with high dose AAI (5 mg/kg/day) (Fig. 4a). The weight loss in GNMT KO male mice (2 mg/kg/day) was the same as that in WT male mice (2 mg/kg/day) (Fig. 4b). Serum ALT levels were significantly increased in the high dose of AAI-treated WT mice and low dose of AAI-treated KO mice, however, the low dose of AAI did not elevate the ALT in WT female mice (Supplementary Fig. S5a,b). Treatments with AAI significantly and dose dependently increased SCr levels in WT female mice (Supplementary Fig. S5c,d). Histological analysis showed that treatment with low dose AAI led to severe renal tubular atrophy in GNMT KO female mice but mild kidney injury in WT mice (Fig. 4c). The kidney injury was severe in both GNMT KO and WT male mice treated with low dose AAI (Fig. 4d). These data suggest that GNMT plays a protective role in AAI-treated female mice. Furthermore, GNMT mRNA expression in liver decreased in WT mice after 3 weeks of high dose AAI exposure (Fig. 4e). NQO1 mRNA expression was increased in GNMT KO mice and remained at the high level after AAI treatment compared to WT control mice (Fig. 4f). Also, compared to WT control female mice, CYP3A44 and CYP3A41 expressions were significantly decreased in GNMT KO control female mice (Fig. 4g,h). The CYP3A44 and CYP3A41 expression levels increased in low-dose AAI-treated WT female mice (Fig. 4g,h). This observation matches the histologically-observed severe renal impairment in low-dose AAI-treated KO female mice and minor renal damage in low-dose AAI-treated WT female mice (Fig. 4c). These results further support that GNMT protects female mice from AAI-induced renal damage via increasing CYP3A44/41 expression.

### Re-expression of hepatic GNMT decreases AAI-induced kidney injury

**Figure 5:**
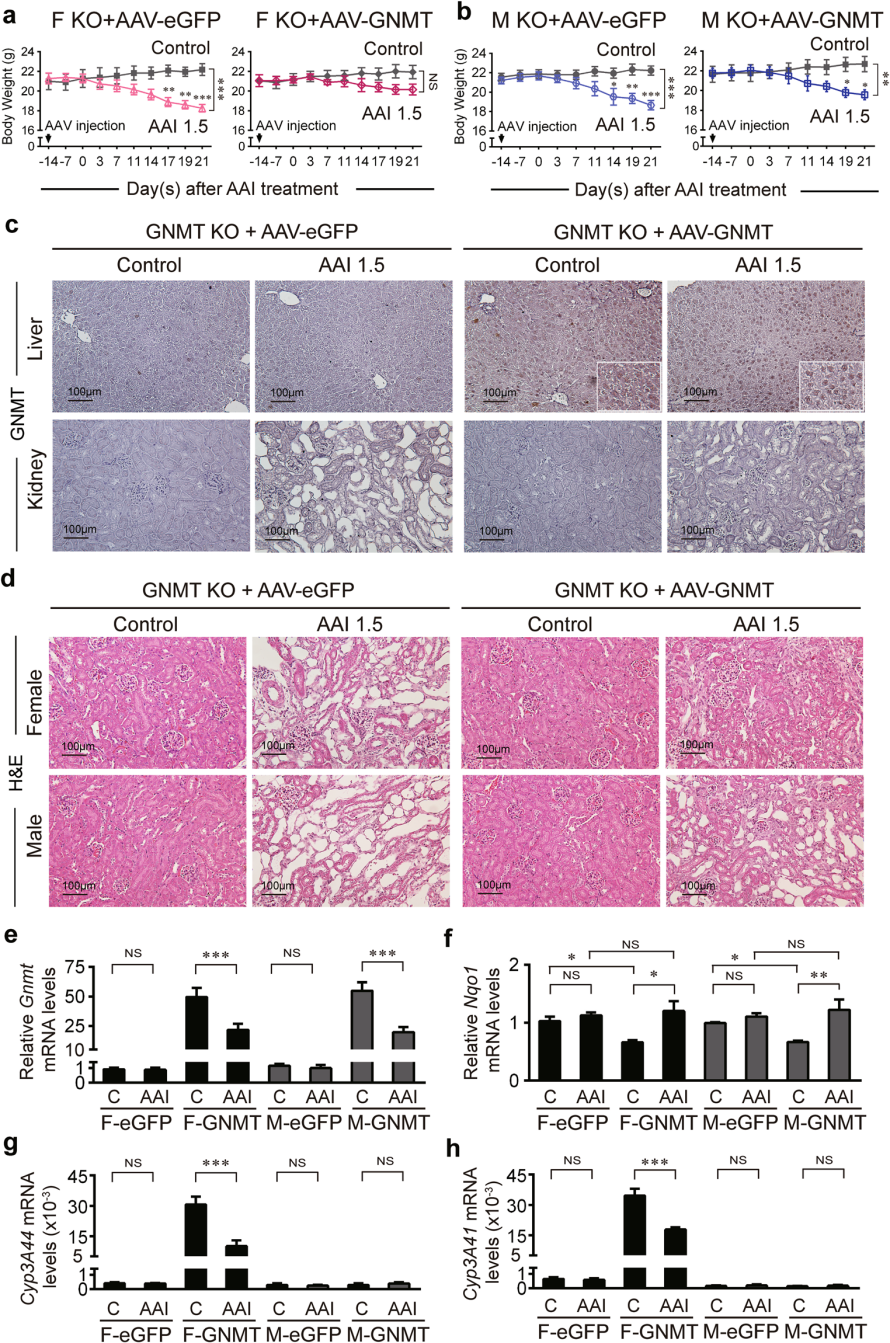
Re-expression hepatic GNMT reduces the AAI-induced nephropathy. GNMT knockout (KO) mice were intravenously injected with AAV-GNMT or AAV-eGFP. At 14 days after AAV injection, all mice were challenged with AAI at a dose of 1.5 mg/kg/day (AAI 1.5), 5 days/week for 3 weeks. (**a**,**b**) Body weight of AAV-GNMT or AAV-eGFP-treated KO mice intraperitoneally injected with AAI or corn oil (vehicle control). Values are presented as the mean ± SEM, n = 4. *p*-values were calculated by two-way ANOVA and Sidak’s multiple comparisons test. (**c**) Representative micrographs of GNMT expression in the liver and kidneys of the AAV-treated KO mice (original magnification ×200). (**d**) Hematoxylin and eosin staining of kidneys in AAV-treated KO mice after 3-week AAI exposure (original magnification ×200). (**e–h**) mRNA level of (**e**) *Gnmt*, (**f**) *Nqo1*, (**g**) *Cyp3A44, and* (**h**) *Cyp3A41* genes from the livers of AAV-treated KO mice after 3-week AAI exposure, as described in (**a**,**b**), were determined by qPCR. Data were normalized by β-actin. (F-eGFP: female KO mice treated with AAV-eGFP, F-GNMT: female GNMT KO mice treated with AAV-GNMT, M: Male) Values are presented as the mean ± SEM, n = 5. *p*-values were calculated using Student’s *t* -test. *p < 0.05, **p < 0.001, ***p ≤ 0.0001.

While GNMT was knocked out in all organs of GNMT knockout (KO) mice, GNMT is overexpressed in the liver and kidneys of human GNMT Tg mice (Fig. 2d and Supplementary Fig. S4e)^38^. To confirm the role of hepatic GNMT in AAI-induced nephropathy, we performed adeno-associated virus (AAV)-based gene therapy to restore GNMT protein in the liver of KO mice and observed the protective ability of GNMT. At 14 days before AAI treatment, KO mice received AAV (type 8)-containing human GNMT by tail vein injection to enhance the expression of GNMT in liver. After the biochemical test for liver function (serum ALT levels) in KO mice declined (Supplementary Fig. S6a,b), both female and male mice were treated with AAI (1.5 mg/kg/day) for 3 weeks. The body weight of KO mice undergoing AAV gene therapy decreased significantly after 3-week AAI exposure (Fig. 5a,b) except the AAV-GNMT treated KO female mice (Fig. 5a; right panel). In all mice after AAV-GNMT gene therapy, GNMT was expressed in the liver but not in the kidneys (Fig. 5c). The serum levels of ALT and creatinine did not elevate by AAI in AAV-GNMT-treated female KO mice (Supplementary Fig. S6c–f). Consistent with these observations, kidney injury decreased in GNMT KO mice after AAV-GNMT gene therapy (Fig. 5d).

The qPCR results showed that, liver GNMT can be restored through AAV-mediated GNMT gene transfer (Fig. 5e) to reduce the NQO1 mRNA level (Fig. 5f) and increase CYP3A44 and CYP3A41 mRNA levels (Fig. 5g,h) in the corn oil-treated (vehicle control) mice. After AAI exposure, the NQO1 was upregulated (Fig. 5e) and CYP3A44 and 3A41 were downregulated (Fig. 5g,h) in AAV-GNMT-treated female KO mice. However, the CYP3A44 and CYP3A41 mRNA levels in AAV-GNMT-treated female KO mice remained higher than that in AAV-eGFP-treated female KO mice after AAI exposure (Fig. 5g,h). In summary, GNMT serves as a protector to prevent kidney injury from AAI toxicity by upregulating CYP3A44 and CYP3A41 in mouse liver. In the absence of GNMT, the kidneys were not protected from damage.

### AAI increases the translocation of GNMT into the nucleus

**Figure 6:**
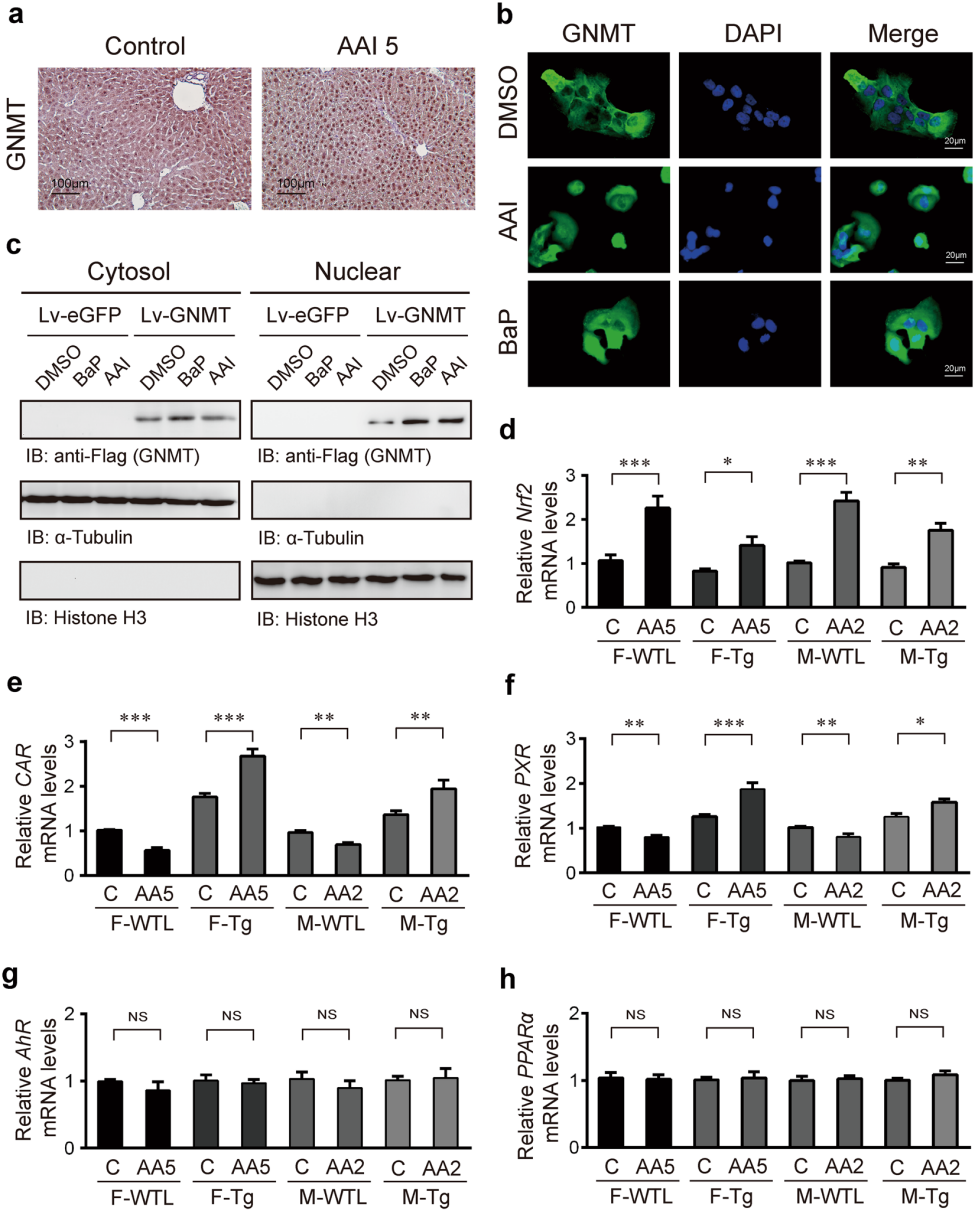
AAI promotes the nuclear localization of GNMT in the liver. (**a**) Immunohistochemical staining for GNMT expression in the liver of the female C57BL/6 mouse intraperitoneally injected with 5 mg/kg bw/day AAI (AAI 5) or corn oil (vehicle control) for 3 weeks (original magnification ×200). (**b**) Immunofluorescent microscopy of fixed Huh7 Lv-GNMT cells stained for GNMT (green, Alexa 488) and DAPI. Huh7 Lv-GNMT cells were treated with 250 μM AAI, 10 μM BaP (positive control) or vehicle (0.01% DMSO, negative vehicle control) for 16 hours (original magnification, ×200). (**c**) Western blot analysis of nuclear and cytoplasmic (non-nuclear) fractions from AAI-, BaP- and DMSO-treated Huh7 Lv-GNMT cells. Histone H3 and α-tubulin were used as loading and purity controls for the nuclear and cytoplasmic fractions, respectively. The full-length blots are presented in Supplementary Fig. S7. (**d–h**) mRNA levels of (**d**) *Nrf2*, (**e**) *CAR*, (**f**) *PXR* **(g)** *AhR*, **(h)** *PPARα* in the liver from AAI- or corn oil-treated hGNMT Tg mice, as described in Fig. 2a and b, were determined by qPCR. (C: mice treated with corn oil; AA2 orAA5: 2 or 5 mg/kg BW/day AAI administration; F: female; M: male; Tg: hGNMT transgenic mice; WTL: wild-type littermates of Tg mice) All qPCR data were normalized to β-actin. Values are presented as the mean ± SEM; n = 4 in each group. *p*-values were calculated using Student’s *t*-test. *p < 0.05, **p < 0.001, ***p ≤ 0.0001.

Notably, strong nuclear staining for GNMT was observed in liver specimens of AAI-treated mice when compared to corn oil-treated mice (Figs 5c and 6a). Using the immunofluorescent staining assay, we clearly observed GNMT nuclear translocation of Huh7-LvGNMT cells by AAI stimulation (Fig. 6b). Once again, western blot analysis confirmed the results of GNMT nuclear translocation in response to AAI exposure (Fig. 6c). But, the functions of nuclear GNMT remain unknown.

Aryl hydrocarbon receptor (AhR), constitutive androstane receptor (CAR), pregnane X receptor (PXR), peroxisome proliferator-activated receptor (PPARα) and nuclear factor-E2-related factor 2 (Nrf2) are well-characterized xenobiotic-activated transcription factors in liver that regulate the induction of drug metabolizing enzymes (DMEs) and transporters such as CYP450s and NQO^53–56^. In our microarray data analysis, the genes significantly up- and down-regulated by AAI were overlaid onto the canonical xenobiotic metabolism pathway by using Ingenuity Pathways Analysis (IPA) program. The activation or inhibition effects of path were predicted by IPA molecule activity predictor (IPA-MAP) tools. The IPA-MAP predictions showed that the Nrf2/NQO1 and AHR signaling pathways were activated and the PXR and CAR signaling pathways were inhibited in the liver of WT female mice treated with 5 mg/kg/day AAI, 5 days a week for 3 weeks (Supplementary Fig. S8). qRCR assays revealed that after AAI exposure, both *Nrf2* mRNA levels in WTL mice and Tg mice were significantly elevated (Fig. 6d), the increased level in the AAI-treated Tg mice, however, was not as high as that in AAI-treated WTL mice (Fig. 6d). The *CAR*/*PXR* mRNA levels were significantly decreased in AAI-treated WTL mice and increased in AAI-treated Tg mice (Fig. 6e,f). However, *AhR* and *PPARα* mRNA levels were not significantly different in all AAI-treated WTL mice and Tg mice (Fig. 6g,h). These results showed that *Nrf2*, *CAR* and *PXR* mRNA levels were associated with GNMT expression upon AAI stimulation, suggesting the involvement of GNMT in the regulation of transcription factors.

### **Nuclear GNMT interacts with*CAR/PXR*or*Nrf2*transcriptions**

**Figure 7:**
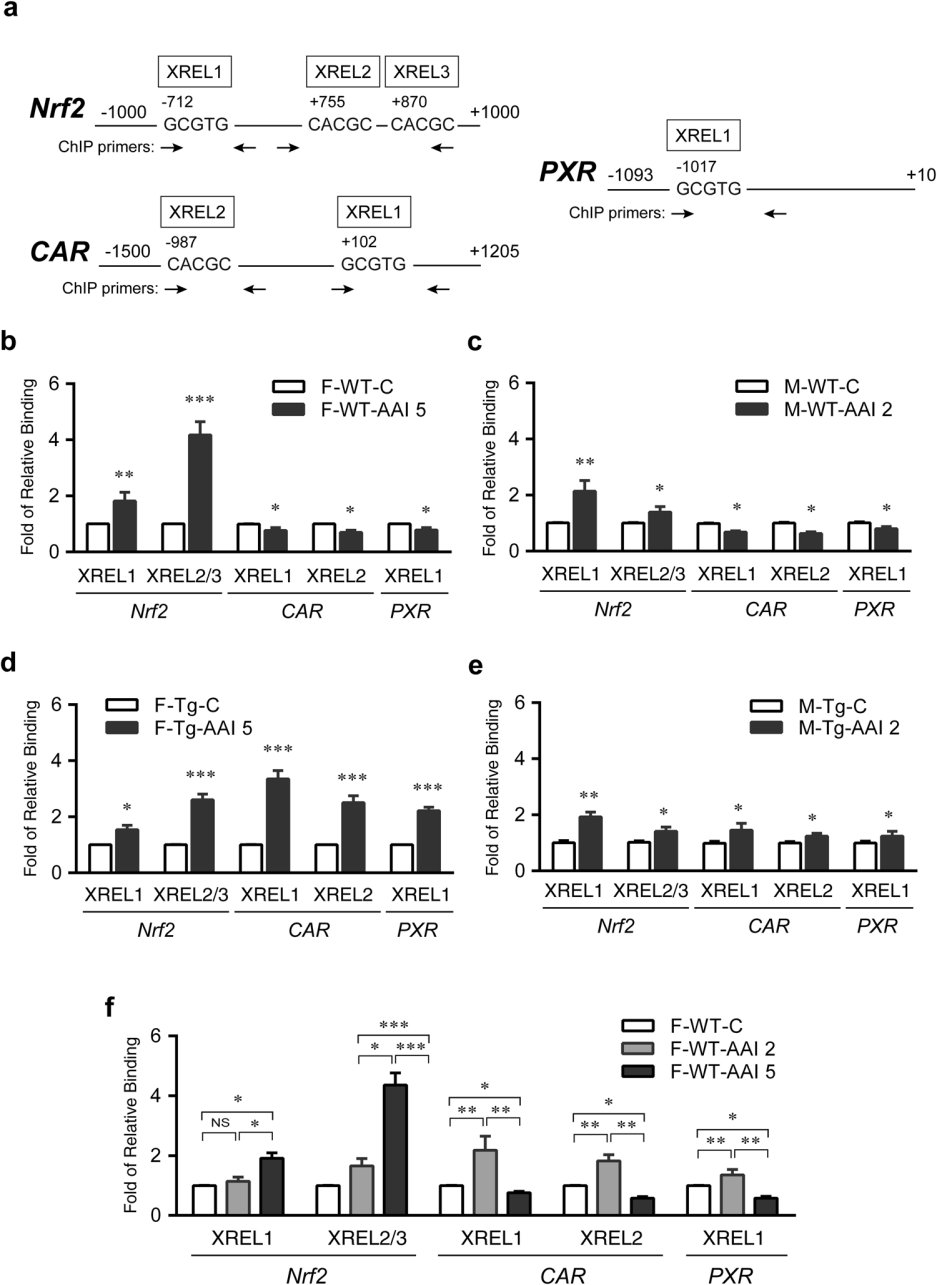
GNMT interacts with the promoters of the genes encoding Nrf2, CAR and PXR, nuclear transcription factors. **(a)** The chromatin was immunoprecipitated with mouse anti-GNMT antibody (14-1) or mouse IgG (negative control). ChIP-enriched DNA was amplified with the primers for XREL1, XREL2 and XREL3 regions on the promoter of *Nrf2*, *PXR*, and *CAR* genes. Schematic represents the upstream promoter binding regions of XREL1, XREL2 and XREL3 in the *Nrf2* (XREL1: −712 to −708; XREL2: +755 to +759; XREL3: +870 to +874)*, CAR* (XREL1: +102 to +106; XREL2: −987 to −983) and *PXR* (XREL1: −1017 to −1013) genes. Horizontal arrows indicate the location of primers used for qPCR in site-specific ChIP assays. Note that the figure is not drawn to scale. **(b–e**) ChIP-qPCR analyses were performed with the liver lysates from AAI- or corn oil (C)-treated WT (**b**, female, F; **c**, male, M) and hGNMT Tg mice (**d**, female, F; **e**, male, M), as described in Figs 1 and 2. (**f**) ChIP-qPCR analyses were performed with the liver lysates from 3-week AAI- and corn oil (C)-treated WT female mice, as described in Fig. 4a (AAI 2 or AAI 5: 2 or 5 mg/kg BW/day AAI administration). The quantity of DNA in the precipitation with GNMT antibody was normalized to the IgG control. Values are presented as the mean ± SEM, n = 4. *p*-values were analyzed using the one-way ANOVA or Student’s *t*-test. *p < 0.05, **p < 0.001, ***p ≤ 0.0001.

To observe whether GNMT interacts with *Nrf2, PXR* and *CAR* genes in the nucleus upon AAI stimulation, chromatin immunoprecipitation (ChIP) assays were performed (Fig. 7a). ChIP results showed that in the liver of AAI-treated WT mice, the fold change in the amount of *Nrf2* promoter DNA pulled down with anti-GNMT antibody (GNMT-*Nrf2*) significantly increased up to 4.2-fold (Fig. 7b,c), whereas the amounts of GNMT-pulled down *CAR* and *PXR* promoter DNAs (GNMT-*CAR* and GNMT-*PXR*) decreased (Fig. 7b,c). In AAI-treated Tg female mice with minor kidney injury, GNMT-*CAR* and GNMT-*PXR* markedly increased (Fig. 7d). Meanwhile, the GNMT-*Nrf2* also increased in AAI-treated Tg female mice, but the level was not as high as that in AAI-treated WT mice (Fig. 7d,b). Interestingly, in male GNMT Tg mice with severe kidney damage, GNMT-*CAR* and GNMT-*PXR* levels slightly increased, but were still lower than that of GNMT-*Nrf2* (Fig. 7e). We next investigated the nuclear GNMT interactions in female wild-type mice after exposure to different concentrations of AAI (low concentration: 2 mg/kg/day versus high concentration: 5 mg/kg/day). The ChIP data indicated that GNMT-*CAR* and GNMT-*PXR* were higher at low AAI concentration than at higher concentration (Fig. 7f), resulting in increases in CYP3A44/3A41 mRNA levels (Fig. 4g and h) and minor kidney injuries (Fig. 4c). The high concentration of AAI increased GNMT-*Nrf2* (Fig. 7f), upregulated *NQO1 mRNA* levels (Fig. 4f) and damaged kidney severely (Fig. 4c). Taken together, these results indicate that hepatic GNMT protect mice against AAI-induced renal injury.

## Discussion

**Figure 8:**
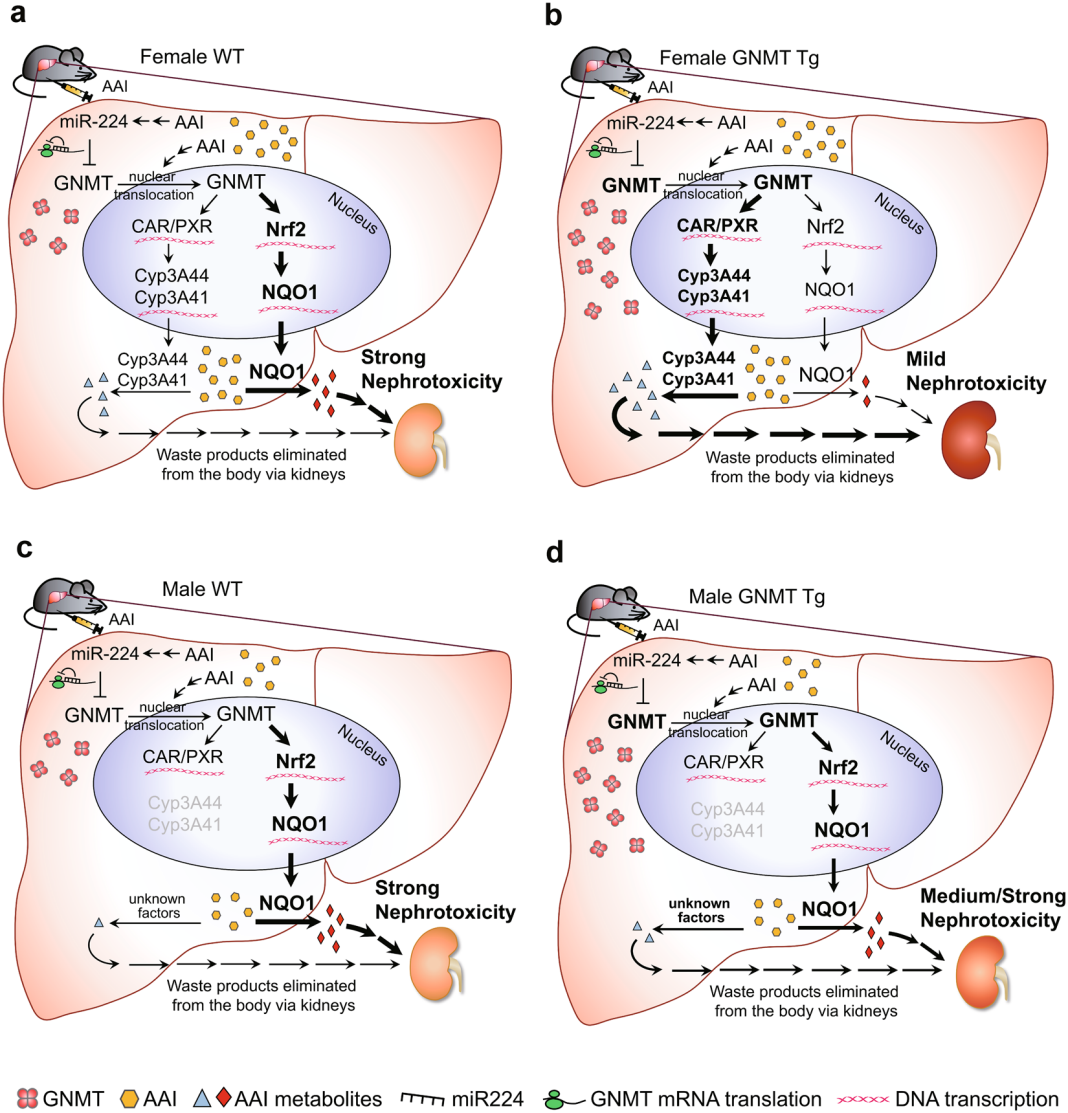
Schematic representation of the role of GNMT in AAI metabolism. The proposed metabolic pathways of AAI regulated by GNMT in liver of 5 mg/kg/day AAI-treated female WT mice **(a)** and GNMT Tg mice (**b**); and in 2 mg/kg/day AAI-treated male WT mice (**c**) and GNMT Tg mice (**d**).

In this study, we revealed that AAI promotes GNMT nuclear translocation and nuclear GNMT can bind to the promoter regions of *NRF2* and *CAR*/*PXR* and enhance subsequent *NQO1* and *CYP3A44/3A41* transcriptions, respectively. The possible pathways of GNMT involvement in AAI metabolism are illustrated in Fig. 8. AAI exposure suppressed cytosolic GNMT expression and induced GNMT nuclear translocation in mouse liver. In WT female mice, AAI exposure increased GNMT-*Nrf2*, resulting in the elevated *NOQ1* transcription, and decreased GNMT-*CAR* and GNMT-*PXR*, resulting in a reduction of CYP3A44/CYP3A41 transcripts. These effects of AAI thus seriously enhanced kidney injury (Fig. 8a). In contrast, in GNMT Tg female mice receiving AAI, high concentrations of GNMT favored *CAR/PXR* transcription in nucleus, CYP3A44/CYP3A41 mRNA levels were thus upregulated, and the kidneys mildly damaged (Fig. 8b). However, in male mice, absence of Cyp3A44/3A41was related to the AAI intolerance, even in the GNMT Tg mice, the AAI-induced nephropathy was barely decreased (Fig. 8c,d). Overall, the difference in the hepatic GNMT expression contributes to the different outcomes.

Here, we verified that exposure to the AAI, as well as BaP^35–37^ and AFB1^38,39^, promotes GNMT nuclear translocation in liver cells. There are three forms of GNMT: monomers, dimers and tetramers, and only GNMT monomers are able to enter the nuclei and interact with chromatin^57^. GNMT dimers were not seen in cytosol and nuclei of control liver tissue cells, but were found in nuclei of liver tissue cells after BaP exposure^37^. Both GNMT monomers and dimers were catalytically and folate-binding inactive^34,58,59^. It is reasonable to suggest that the main effect of nuclear-translocated GNMT is transcriptional regulation.

In AAI-treated animals, we observed that male mice did not tolerate high dose of AAI (Fig. 1a,b) and the weight loss in male mice was much more than that in female mice after 3-week 2 mg/kg AAI exposure (Fig. 4a,b). Kidneys from AAI-treated group were paler but not smaller than control group (Supplementary Fig. S1a). Also, the fibrosis was not observed in 3-week AAI-treated kidneys. These findings confirmed previous publications^11,40,41^. It has been reported that, after C57BL/6 female and male mice treated with 3 mg/kg of AAI once every 3 days for 6 weeks and then maintained for another 6 weeks, the weight reduction in AAI-treated male mice is 2-fold greater than that in female mice and kidneys from AAI-treated animals are smaller and paler than control. In addition, single or double dose of 2.5 mg/kg AAI treatment fails to induce fibrosis in 4 weeks^41^. It is conceivable that the duration of our experiments may be too short to observe the shrunken kidneys and renal fibrosis in AAI-treated mice.

In the literature, human CYP1A2, CYP2C9, CYP3A4 and CYP1A1 were the major enzymes contributing to AAI oxidation in the human liver, while CYP2C and CYP1A were the most important in rat and mouse liver^13,15^. In our microarray data, CYP3A44 was the most significantly downregulated gene in female mouse liver after AAI exposure. CYP3A44 and CYP3A41 are expressed abundantly in female mice, with almost no expression in male mice (Fig. 3c,d)^50^. Our findings suggest that CYP3A44/3A41 regulated by GNMT is highly related to reduced AAI nephropathy in mice, although the catalytic activities of mouse CYP3A44/CYP3A41 towards AAI have never been reported. Human CYP3A4 is the homologue of mouse CYP3A44/CYP3A41, a protein mainly expressed in the liver and small intestine and responsible for the metabolism of many drugs. Women have higher CYP3A4 activity than men^51,60^; thus, many drugs metabolized by CYP3A4 clear faster in women than men^52,61^. In the clinical setting, it is very difficult to know whether and how much AAs has been consumed by a kidney-impaired patient. Also, the limitation of this work is the confirmation of AA metabolism through liver specimen evaluation. We hypothesize that men are less tolerant of AAI toxicity than women, and that acute renal injury warned men to stop self-medication or intake of illegal AAs-contaminated herbal remedies. The continuous production of oncometabolites from AA intake in women may be the reason for the higher incidence of upper tract urothelial cancer in Asia, especially in Taiwan^62^.

The kidney injury induced by AAI may be a consequence or independent from liver damages. In our short 3-week *in vivo* study, histological examination did not show liver damage. However, microarray analysis showed that thousands of genes were significantly changed in liver after AAI exposure. In addition to the xenobiotic metabolism pathway, the signal transduction pathways affected by AAI, according IPA analysis, include inflammation response, cytokine/chemokine signaling, immune cell (dendritic cells, T cells, B cells) activation, communication between innate and adaptive immune cells, cholesterol biosynthesis, hepatic fibrosis/hepatic stellate cell activation and acute phase response signaling pathways. Changes of the gene expressions or signal transduction pathways in liver may affect kidneys in a variety of ways: the metabolites produced in liver, through the release of cytokines or other proteins by injured liver, through the activation of immune cells, though abnormal amounts of enzymes, etc^63–66^. However, our results do not exclude the possibility that AAI may directly cause the kidney damage. Indeed, it has been reported that AAI may directly cause renal toxicity by the *in vitro* evidence in which AAI damages the mitochondrial transition permeability and induces apoptosis in HK2 cells^67^. Further studies are needed to elucidate underlying mechanisms.

The expression of GNMT in kidney is much lower than that in liver. CYP1A1, 1A2 and CYP2C9 are not expressed in human kidney, whereas the CYP3A4 expression in kidney is highly variable and remains to be elucidated^68^. In mice, CYP1A1, CYP1A2 and CYP3A44/3A41 are almost not expressed in kidney^50^. In contrast, NQO1 is highly expressed in renal tubulars and plays a major role in renal AAI nitroreduction^17,18,69^. This is a possible pathway of AAI metabolism regulated by GNMT in kidney and may thus contribute to AAI-induced nephrotoxicity and DNA-adduct formation. This may explain why GNMT does not protect kidney from the damage.

In GNMT knockout mice, the absence of GNMT resulted in accumulated free methionine, a 35-fold increase of SAM and a 100-fold increase of SAM/SAH ratio in liver^33^. However, the hepatic glutathione (GSH) levels are similar in GNMT KO and WT mice in spite of the significant reduction in SAM-dependent methylation reactions^70^. It has been reported that elevated concentration of SAM reduces CYP2E1^71^ and CYP39A^72^, but markedly induces CYP4A^73^ in GNMT KO mice. These CYP450s participate in hepatic lipid metabolism and xenobiotic detoxification^70–73^. Interestingly, in our microarray data, CYP4A were significantly induced up to 4- to 6-fold by AAI. The abnormal expressions of these CYP450s may contribute to AAI toxicity in kidney in the absence of GNMT.

In conclusion, we successfully developed the *in vivo* GNMT transgenic, knockout and AAV-type 8 GNMT gene transfer mouse models for AA nephropathy. GNMT protects mice from AAI-induced kidney injury by increasing *CAR/PXR/CYP3A44/3A41* transcription and decreasing *Nrf2/NQO1* transcription in female hepatocytes. Furthermore, sex differences in hepatic xenobiotic metabolism affect kidney function.

## Methods

### Animals

Six- to eight-week-old female and male C57BL/6 mice were obtained from the National Laboratory Animal Center (Taipei, Taiwan). The GNMT knockout mice in C57BL/6 background^74^, GNMT transgenic mice in FVB/B6 background^38^ and their littermate wild-type control were generated and maintained in specific pathogen-free conditions in accordance with the regulations at the Animal Center, Kaohsiung Medical University (KMU). Mice were treated with 2 or 5 mg/kg/day AAI (Sigma-Aldrich, St. Louis, MO, USA) or corn oil (vehicle control) by intraperitoneal (i.p.) injection, 5 days per week for 3 weeks and then were humanely euthanized by CO_2_. Mouse serum were collected from tail vein before and after AAI treatment. Serum levels of ALT, creatinine and BUN were detected by FUJI DRI*-*CHEM 4000i analyzer (FUJIFILM Corp. Tokyo, Japan). Mouse liver and kidneys were also collected to future experiments. All experiments were performed in accordance with relevant guidelines and regulations. All animal work was approved by the Institutional Animal Care and Use Committee of KMU (IACUC approval no.: 104075, 104093).

### Cell lines

The HCC cell line, Huh7, was maintained in Dulbecco’s Modified Eagle’s medium (DMEM) (Gibco BRL, Grand Island, NY, USA) with 10% heat-inactivated fetal bovine serum (HyClone, Logan, UT, USA), 2 mM/L L-glutamine, 0.1 mM/L nonessential amino acids, 100 U/ml penicillin and 100 μg/ml streptomycin in a humidified incubator with 5% CO_2_ at 37 °C. The pLKO-AS3w-puro expression vector was purchased from the National RNAi Core Facility (Academia Sinica, Taipei, Taiwan). Lentiviral vector express GNMT (pLKO-AS3w-GNMT) was constructed according to manufacturer’s recommendation. The pLKO-AS3w-eGFP-puro plasmid was used as a negative control. The plasmid DNA was transfected into Huh7 cells (Huh7-LvGNMT or Huh7-eGFP). After 48 hours, transfected cells were then selected with 1 μg/ml puromycine (Sigma-Aldrich, St. Louis, MO, USA) to establish the GNMT overexpression stable clone for future experiments. For *in vitro* treatments, AAI was dissolved by DMSO in 250 mM for treatment 1:1000 in cell culture medium.

### GNMT promoter luciferase reporter assays

Huh7 GNMT promoter-luciferase (H7GPL) cells^75^ were seeded at 96-well plates, 5 × 10^3^ cells in each well. H7GPL cells were treated with 2.5, 5, 25, 50, 100, 250, 500 μM AAI or 0.1% DMSO (vehicle negative control) in duplicate for 16 hrs. Treated H7GPL cells were lysed for luciferase activity measurement by using the One-Glo^TM^ Luciferase Assay System (Promega, E6120) and detected in a luminometer (BioTek Ins., Synergy HT). The reporter luciferase activity were normalized by cell density measured by adding alamarBlue® reagent (Thermo Scientific/Pierce Biotechnology., Rockford, lL, USA). Values were presented as the relative luciferase activity, fold increase over vehicle control.

### RNA extraction and real-time quantitative PCR

Total RNA from mouse liver and kidney specimens was extracted by TRIzol reagent (Invitrogen Corp., Carlsbad, CA, USA) according to the manufacturer’s protocol. RNA concentration was quantified by absorbance at 260 and 280 nm using a spectrophotometer (Nanodrop ND1000, Thermo Fisher Scientific). 5 μg RNA was reverse-transcribed into cDNA using Super Script II Reverse Transcriptase Kit (Invitrogen Corp., Carlsbad, CA, USA). The real-time quantitative PCR (qPCR) analysis was performed in the ABI StepOne Plus^TM^ (Applied Biosystems) by using SYBR Green Master Mix (Thermo Scientific). The qPCR conditions were optimized as 95 °C for 10 min and 40 cycles of 95 °C for 10 sec, 60 °C for 10 sec, and 72 °C for 20 sec followed by routine melting and cool conditions. All mRNA levels were normalized by the β-actin mRNA level and results are shown as fold changes compared with the WT control group. The primer pairs used for qPCR were listed in Supplementary Table S1.

### Microarray analysis

The microarray data are available at https://www.ncbi.nlm.nih.gov/geo/query/acc.cgi?acc=GSE101530. For microarray hybridization, total liver RNA was isolated from four groups of animals, AAI- or corn oil-treated female and male C57BL/6 wild type mice, using RNeasy Mini Kits (Qiagen Inc., Valencia, CA, USA). In each group, pooled RNA was prepared by mixing the same amount total RNA from 5 mice. The microarray hybridization was performed by Welgene Biotech, Co., Ltd. (Taipei, Taiwan) using SurePrint G3 Mouse GE 8 × 60 K Microarray kit (Agilent Technologies, CA, USA). Genes that were significantly up- or downregulated by more than 2-fold were subjected to GO enrichment analysis using the cluster Profiler software. Normalized intensities were transformed to gene expression log_2_ ratios between the control and AAI treatment groups. The genes with log_2_ ratio ≥ 1 or  ≤ −1 and *p*-value < 0.05 were collected for further analysis. The analyses of gene expression patterns, canonical pathways and directional predictions were generated through the use of Ingenuity Pathway Analysis (IPA^®^, QIAGEN Inc., https://www.qiagenbioinformatics.com/products/ingenuity-pathway-analysis/).

### AAV8-GNMT generation

The cDNA sequence of the human GNMT was amplified by PCR using Expand HiFi Taq polymerase (Roche, Mannheim, Germany), cloned into the pAAV-MCS plasmid under the transcriptional control of the cytomegalovirus enhancer/promoter to generate plasmids pAAV8-mGNMT. The AAV8-eGFP plasmid for the preparation of control AAV vector was a gift from Dr. Tao (IBMS, Academia Sinica, Taiwan). Recombinant AAV8 vector was generated by a standard calcium phosphate transfection method in HEK293 cells by using a three plasmid, helper virus-free packaging method and purified on a CsCl gradient. Eight-week-old Gnmt−/− mice were treated with a tail vein injection of AAV-GNMT (1 × 10^12^ vg/ml) or AAV-eGFP control.

### Immunohistochemistry

Formalin-fixed paraffin-embedded mouse organ samples that underwent AAI treatment were stained with hematoxylin and eosin (H&E) or the primary mouse anti-GNMT antibody (14-1, YMAC Bio Tech, Taiwan). N-Histofine^®^ Mousestain kit (Nichire Biosciences Inc., Tokyo, Japan) was used for IHC staining with a mouse primary antibody on paraffin-embedded mouse tissue sections. The detail procedures are listed in Supplementary material and methods. Microscopy was used to observe the phenomena of kidney injury after AAI exposure.

### Western blot analysis

25~30 μg cell lysate proteins were separated by 10% sodium dodecyl sulfate polyacrylamide gel electrophoresis (SDS–PAGE) and subsequently transferred to PVDF membranes. After blocking, membranes were probed with primary antibodies, followed by incubation with a peroxidase-conjugated goat-anti rabbit or anti mouse antibody and developed with a chemiluminescent substrate (Immobilon Western, Millipore Corp, MA, USA). The primary antibodies used in this study were: rabbit anti-FLAG (F7425, Sigma-Aldrich), mouse anti-GNMT (14-1, YMAC Bio Tech, Taiwan), mouse anti-β-actin (AC-15, Sigma-Aldrich), rabbit anti-Histone H3 (3263-100, Bio Vision). The experiments were repeated at least three times.

### Nuclear/cytosol fractionation

Huh7-LvGNMT cells were treated with 50 or 250 μM AAI, 10 μM B(a)P (positive control) and DMSO (vehicle negative control, the final concentration was 0.1%) for 16 hrs. Separation of nuclear extract from the cytoplasmic fraction from treated Huh7-LvGNMT cells was performed using Nuclear/Cytosol Fractionation Kit (K266-100, BioVision Inc, CA, USA). The extracted nuclear and cytoplasmic protein fractions were analyzed by SDS-PAGE and western blot assay.

### Chromatin immunoprecipitation

Chromatin immunoprecipitation (ChIP) assay was performed using the Magna ChIP^TM^ G Tissue Kit (Millipore Corp, Billerica, MA, USA) according to the manufacturer’s protocol. Briefly, chopped mouse liver tissues were fixed by 1% formaldehyde for protein-DNA cross-linking. The protein-DNA cross-links was collected and then sonicated to an average size of 500~1000 bp. Next, the chromatin was immunoprecipitated with mouse anti-GNMT antibody (14-1) or mouse IgG (negative control) for 16 hrs and the DNA purification were performed by the EZ-Magna ChIP A/G Kit. 2 μl of ChIP-enriched DNA was used as template for 35 cycles of quantitative PCR amplification with designated primers (Supplementary Table S2). Specific primers were used to amplify the promoter regions (XREL1, XREL2 or XREL3) of *CAR, Nrf2, and PXR* genes. The results were represented as fold changes compared with the control group.

### Statistical analysis

Statistical analysis was performed by using GraphPad Prism 6 software (La Jolla, CA, USA). The *p* < 0.05, *p* < 0.001, and *p* ≤ 0.0001 were considered to be statistically significant in this study.

## Electronic supplementary material

Supplementary Information
