# Supplementary material for: Glycine N-methyltransferase inhibits aristolochic acid nephropathy by increasing CYP3A44 and decreasing NQO1 expression in female mouse hepatocytes
Source: Sci Rep. 2018 May 3;8:6960. doi: 10.1038/s41598-018-22298-6 (PMC5934382; doi:10.1038/s41598-018-22298-6)
Supplement: Supplementary file 1 — Supplementary Information [file 41598_2018_22298_MOESM1_ESM.pdf]

Supplementary Information

for

Glycine N-methyltransferase inhibits aristolochic acid nephropathy by increasing CYP3A44 and decreasing NQO1 expression in female mouse hepatocytes

Ming-Min Chang[1,2], Chang-Ni Lin[1], Cheng-Chieh Fang[1], Marcelo Chen[1, 3, 4], Peir-In Liang[5], Wei-Ming Li[6~8], Bi-Wen Yeh[7,8], Hung-Chi Cheng[9], Bu-Miin Huang[2], Wen-Jeng Wu[1, 7, 8, 10, 11], Yi-Ming Arthur Chen[1, 11]

[1]Center for Infectious Disease and Cancer Research (CICAR), Kaohsiung Medical University, Kaohsiung, Taiwan. [2]Present Address: Department of Cell Biology and Anatomy, College of Medicine, National Cheng Kung University, Tainan, Taiwan. [3]Department of Urology, Mackay Memorial Hospital, Taipei, Taiwan. [4]Department of Cosmetic Applications and Management, Mackay Junior College of Medicine, Nursing and Management, Taipei, Taiwan. [5]Department of Pathology, Kaohsiung Medical University Hospital, Kaohsiung Medical University, Kaohsiung, Taiwan. [6]Pingtung Hospital, Ministry of Health and Welfare, Executive Yuan, Pingtung, Taiwan. [7]Department of Urology, School of Medicine, Kaohsiung Medical University, Kaohsiung, Taiwan. [8]Department of Urology, Kaohsiung Medical University Hospital, Kaohsiung, Taiwan. [9]Department of Biochemistry and Molecular Biology, College of Medicine, National Cheng Kung University, Tainan, Taiwan. [10]Department of Urology, Kaohsiung Municipal Ta-Tung Hospital, Kaohsiung, Taiwan. [11]Graduate Institute of Medicine, College of Medicine, Kaohsiung Medical University, Kaohsiung, Taiwan. Correspondence and requests for materials should be addressed to Y.-M. A. C. (email: arthur@kmu.edu.tw)

Table of contents

Figure S1. Representative the gross morphology and weights of kidneys and livers from AAI-treated C57BL/6 wildtype mice. ……………………………..………... 3

Figure S2. The luciferase reporter assay for studying the interaction of AAI and promoter region of *GNMT* gene. ………………………………………… 4

Figure S3. Liver and kidney miR-224 expression in AAI-treated wild-type and GNMT KO mice. ……………………………...………………………..……… 5

Figure S4. Serum creatinine and ALT of hGNMT transgenic mice. *Gnmt*, *Nqo1* and *Cyp1A2* mRNA levels in the kidney of AAI- or corn oil-treated hGNMT transgenic mice. ………………………………………..……………… 6

Figure S5. Serum creatinine and ALT of GNMT knockout mice at the day before and after 3-week AAI treatment. …………………………...…..……………..…... 7

Figure S6. Serum creatinine and ALT of GNMT KO mice at the day before and after AAV-based gene therapy with GNMT gene. ……………….…………..… 8

Figure S7. Full-length images of the western blots illustrated in Figure 6c. ….............. 9

Figure S8. The directional effect predictions of the xenobiotic metabolism pathway affected by AAI in wild-type female mice. ………………...……….......... 10

Table S1. Primer sequences used for qPCR analysis………………………..……….... 11

Table S2. Primer sequences used in ChIP Assay……………………………..………. 12

Supplementary Methods……………………………………………………......... 13

Supplementary Figures

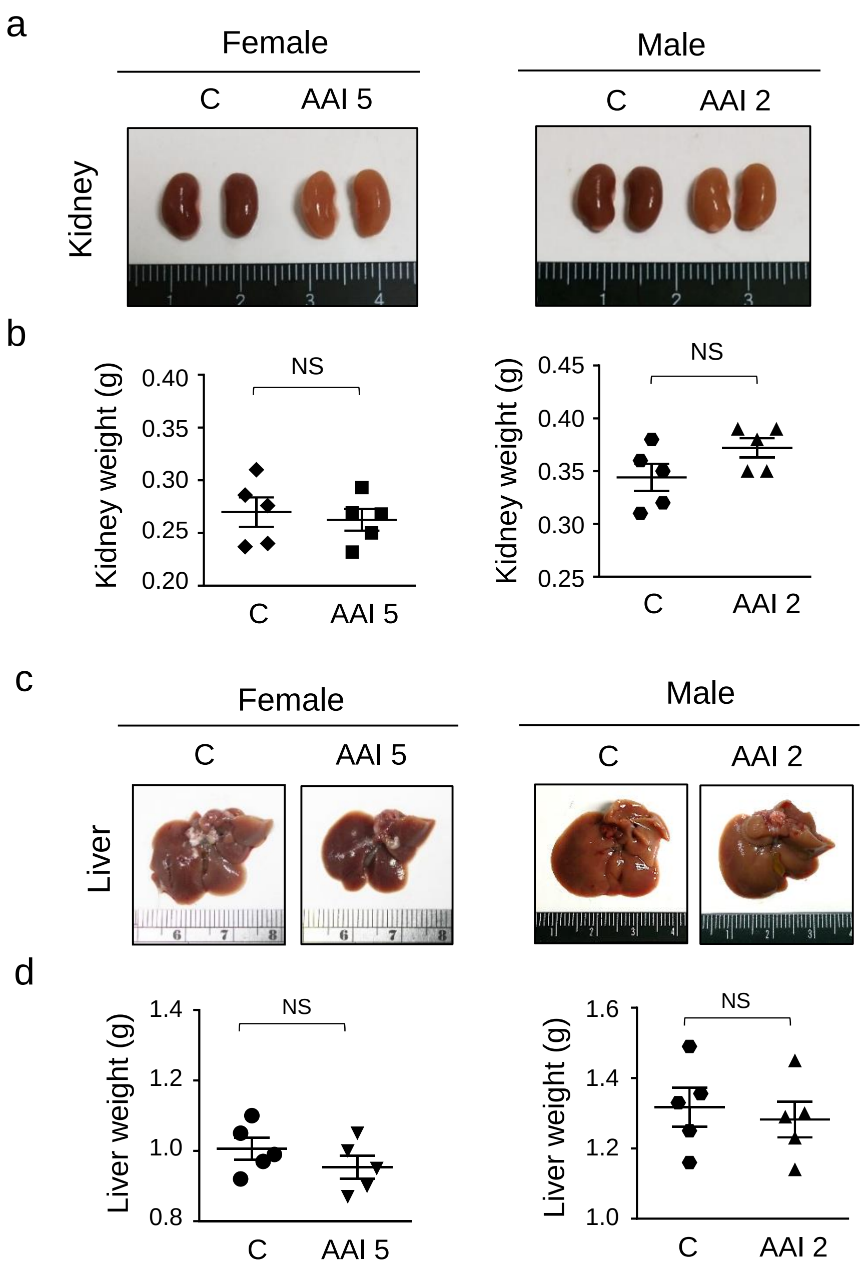

Supplementary Fig S1. Representative the gross morphology and weights of kidneys (**a, b**) and livers (**c, d**) from AAI- or corn oil-treated C57/BL6 wildtype mice as mentioned in Figure 1. AAI 2 or AAI 5: mice were dosed intraperitoneally with 2 or 5 mg/kg body weight/day AAI. C: vehicle (corn oil) control. All data are presented as the mean ± SEM; n = 5 in each group. *p*-values were calculated by Student's *t*-test. NS, not significant.

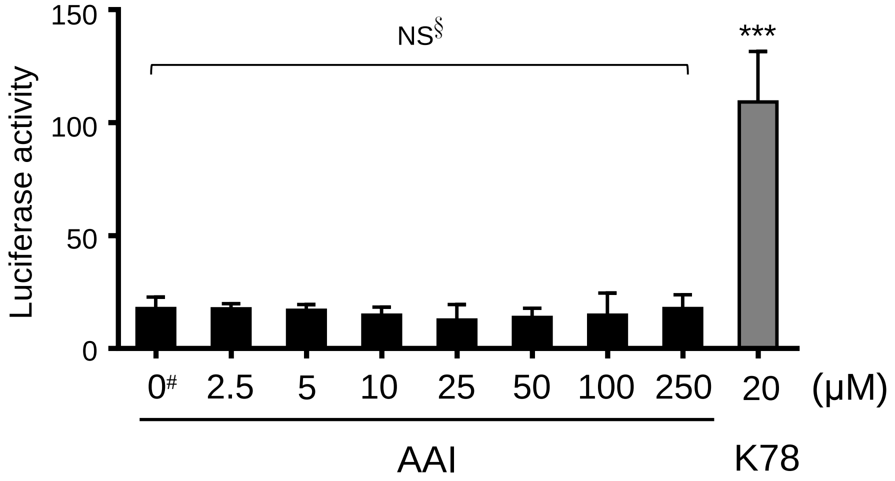

Supplementary Figure S2. The luciferase reporter assay for studying the interaction of AAI and promoter region of *GNMT* gene. Transformed Huh7 cells treated different concentrations of AAI for 16 hours and then performed the luciferase reading. K78 is a chemical compound as a positive control to bind with the promoter region of GNMT. Data were collected from 4 independent experiments (n=4). Values represented as the mean ± SEM; § *p*-values were calculated by one-way ANOVA and Tukey's multiple comparisons test. NS, not significant. #The AAI 0 μM group was treated with DMSO (vehicle negative control). K78 group was compared with the vehicle control, *p*-value were calculated by Student's *t*- test, ***, p<0.0001.

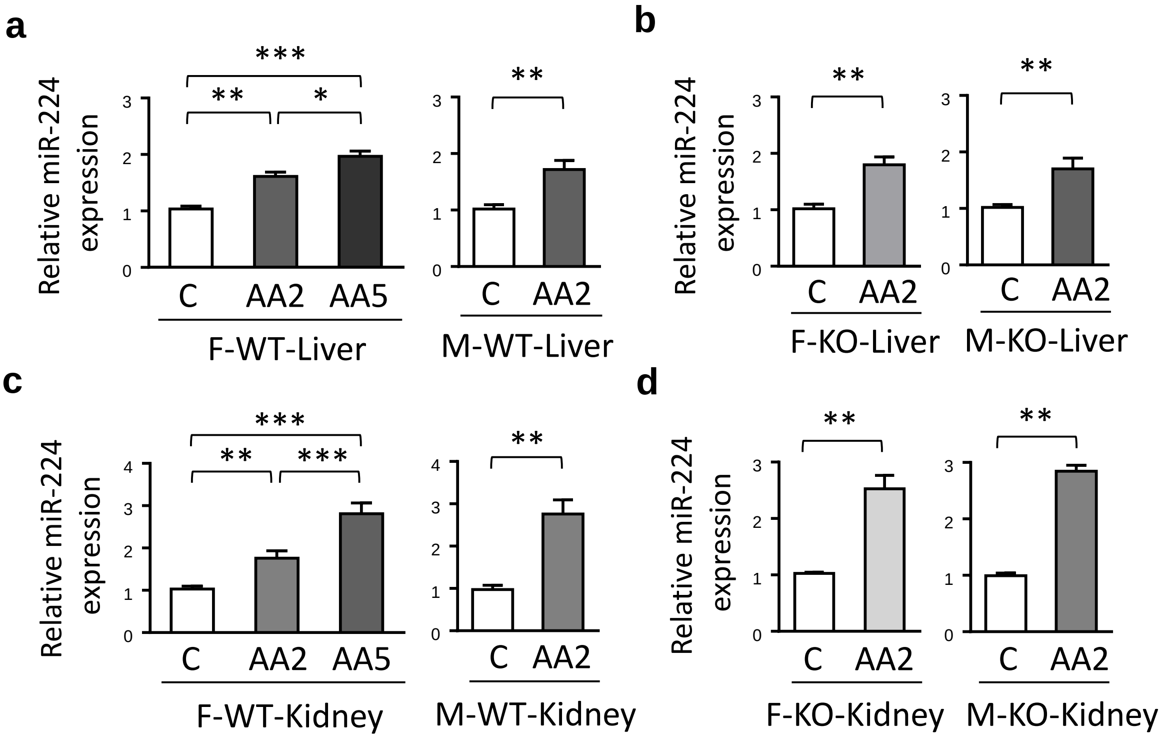

Supplementary Figure S3. Liver and kidney miR-224 expression in AAI-treated wild-type and GNMT KO mice. The relative miR-224 levels were determined using qRT-PCR. sno234 was used as a reference control. Fold-changes were calculated using the $2^{-\Delta\Delta Ct}$ method. C, corn oil vehicle control; AA2 or AA5, mice were dosed intraperitoneally with 2 or 5 mg/kg/day AAI; F, female; M, male; WT, wild-type mice; KO, GNMT KO mice. All liver and kidneys were from the same mice in the Figure 3 (N=5). Representive data were the mean ± SEM. *p*-values were calculated by one-way ANOVA and Tukey's multiple comparisons test or Student's t test. *p<0.05, **p<0.01, ***p<0.001, NS, not significant.

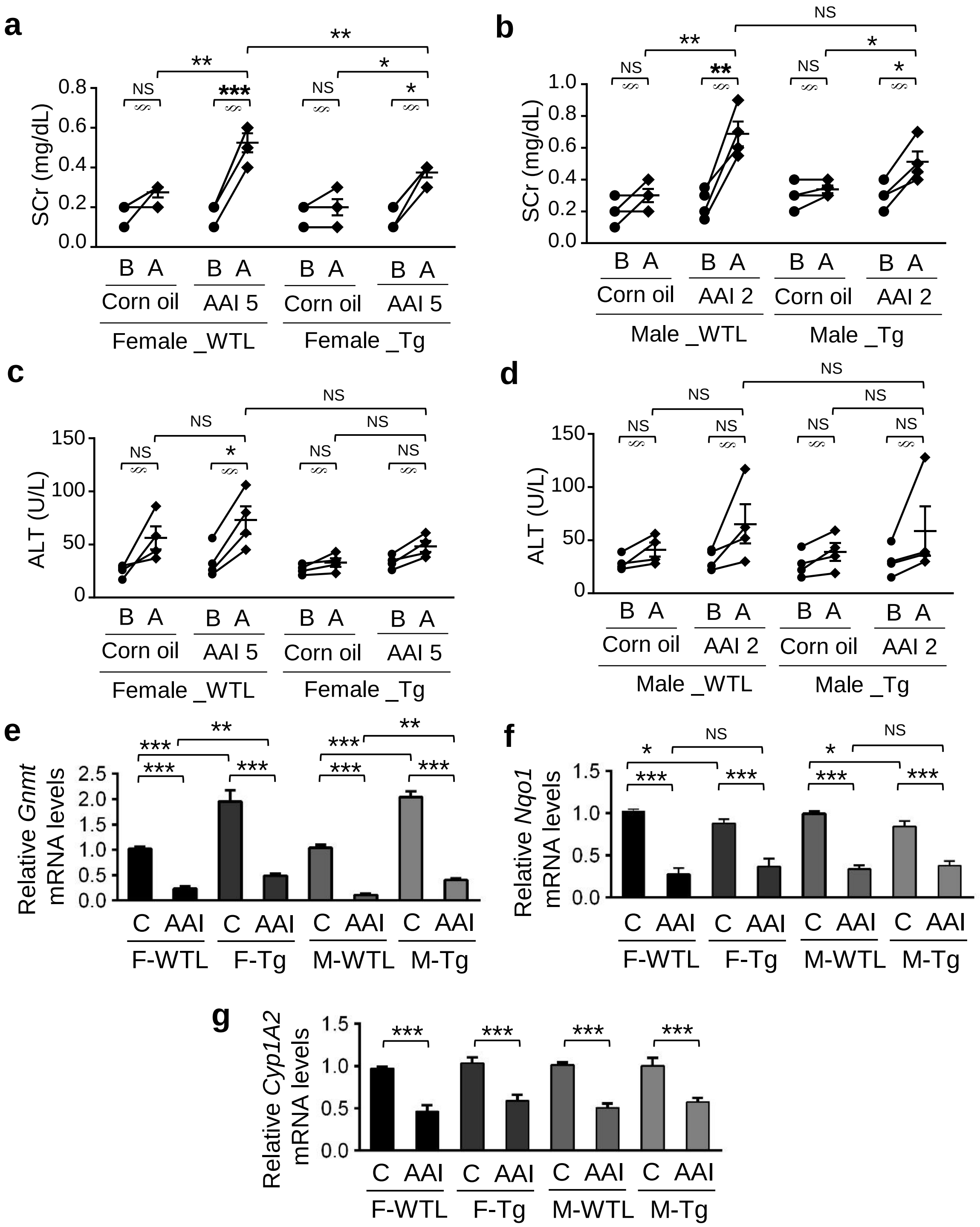

Supplementary Figure S4. Serum levels of creatinine (SCr) (**a, b**) and ALT (**c**, **d**) were measured from female and male hGNMT transgenic mice (Tg) and their wild-type littermate control (WTL) at the day before (B) and after (A) the 3-week AAI treatment, as described in Figure 2. AA2 or AA5, mice were dosed intraperitoneally with 2 or 5 mg/kg/day AAI. (**e-g**) mRNA levels of *Gnmt* (**e**), *Nqo1* (**f**) and *Cyp1A2* (***g***) genes in the kidney of AAI- or corn oil-treated mice, as described in Figure 2. F: female; M: male; C: corn oil vehicle control. All data were represented as the mean ± SEM. ∽ The before (B) and after (A) treatment biochemistry parameters from the same mouse were analyzed by Paired t test. *p*-values for the comparison of biochemistry parameters from AAI- and corn oil-treated mice were calculated by Student's *t*-test. *p<0.05, **p<0.01, ***p<0.001, NS, not significant.

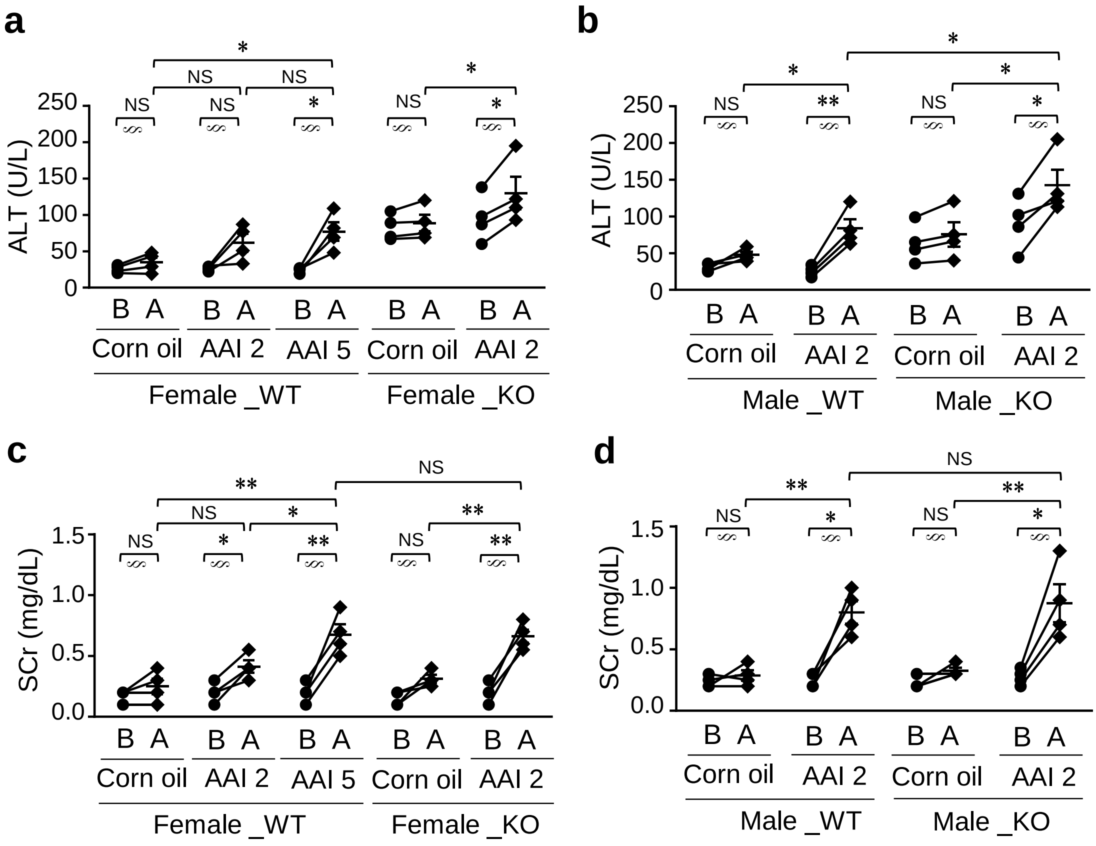

Supplementary Figure S5. Serum levels of ALT **(a, b)** and creatinine (SCr) (**c, d**) were measured from female and male GNMT knockout (KO) or wild-type (WT) mice at the day before and after the 3-week AAI treatment, as described in Figure 4. AA2 or AA5, mice were dosed intraperitoneally with 2 or 5 mg/kg/day AAI. F: female; M: male. All data were represented as the mean ± SEM. ∽ The before (B) and after (A) treatment biochemistry parameters from the same mouse were analyzed by Paired t test. *p*-values for the comparison of biochemistry parameters from AAI- and corn oil-treated mice were calculated by Student's t test. *p<0.05, **p<0.01, ***p<0.001, NS, not significant.

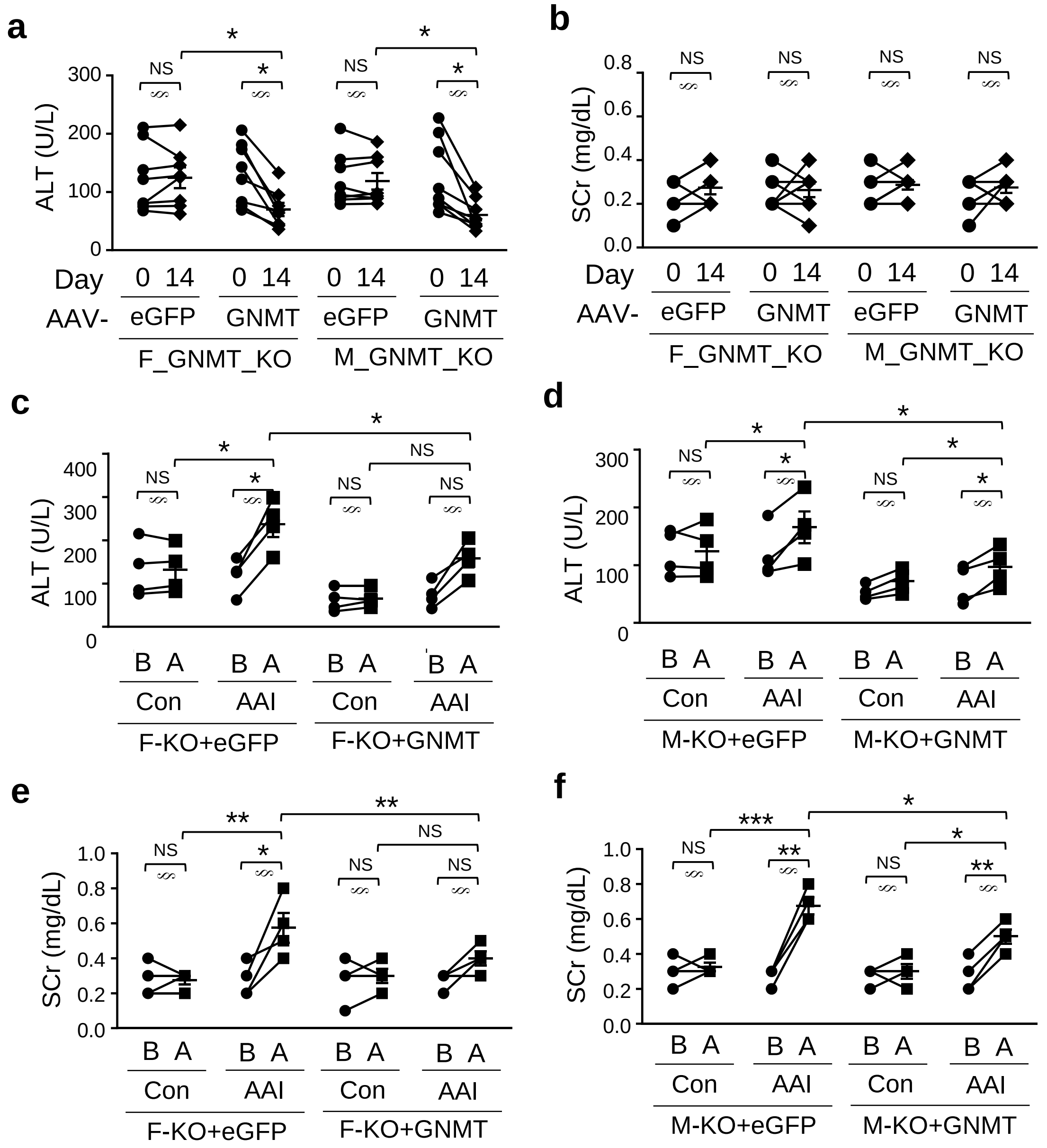

Supplementary Figure S6. Serum levels of ALT (**a**) and creatinine (SCr) (**b**) were measured from female (F) and (**b**) male (M) GNMT KO mice at the day before (0) and after (14) AAV-based gene therapy with GNMT gene or eGFP control as described in Figure 5. (**c-f**) Serum ALT levels and SCr were measured from AAV-treated GNMT KO female (**c, e**) or male (**d**, **f**) mice (eGFP or GNMT) at the day before and after the 3-week AAI treatment, as described in Figure 2. AAI, mice were dosed intraperitoneally with 1.5 mg/kg/day AAI, as described in Figure 5. All data were represented as the mean ± SEM. ∽ The before (B) and after (A) treatment biochemistry parameters from the same mouse were analyzed by Paired t test. *p*-values for the comparison of biochemistry parameters from AAI- and corn oil-treated mice were calculated by Student's *t*-test. *p<0.05, **p<0.01, ***p<0.001, NS, not significant.

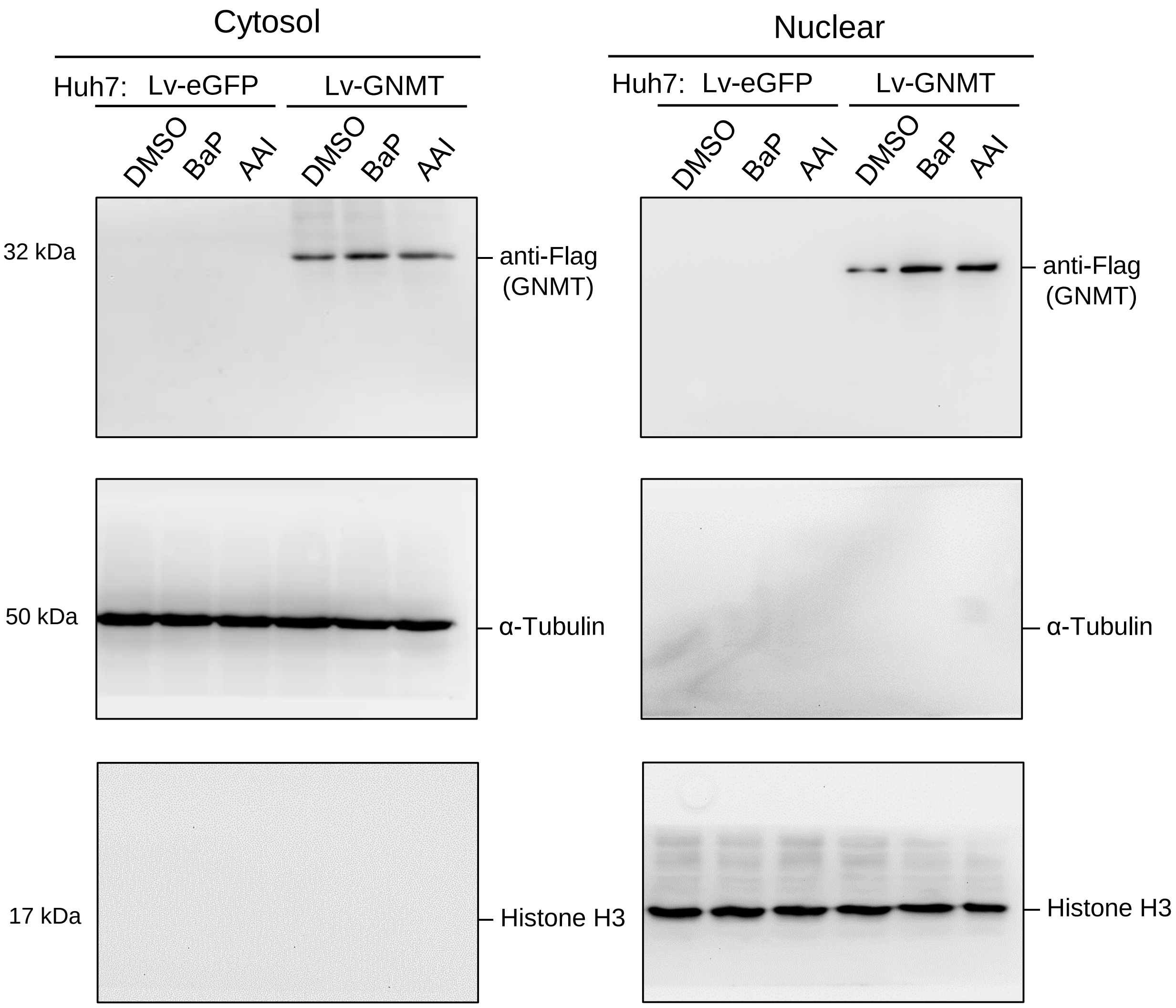

Supplementary Figure S7. Full-length images of the western blots illustrated in Figure 6c.

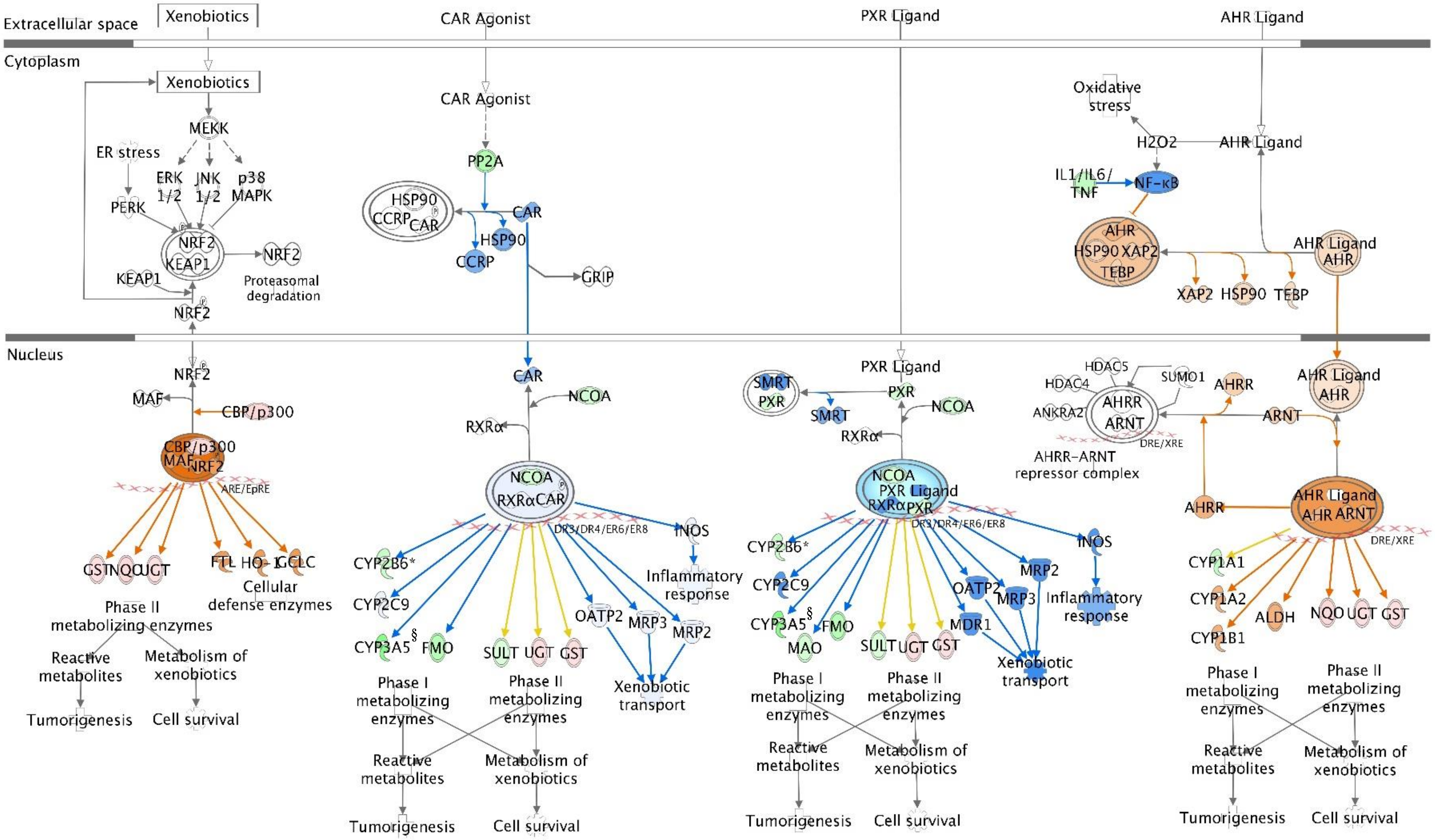

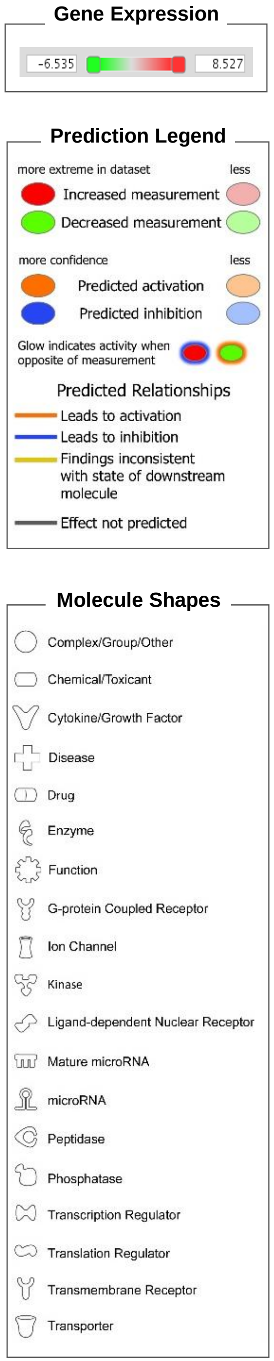

Supplementary Figure S8. The directional effect predictions on the xenobiotic metabolism pathway affected by AAI in wild-type female mice were generated through the use of Ingenuity Pathways Analysis (IPA, QIAGEN Inc., https://www.qiagenbioinformatics.com/products/ingenuity-pathway-analysis). The genes significantly regulated by AAI were mapped onto the canonical xenobiotic metabolism pathway. The activation or inhibition effects of path were predicted by IPA molecule activity predictor (MAP) overlay tools. Red-Green color indicates the level of gene expression. Orange color indicates the activation effect, whereas blue color indicates inhibition effect. Yellow color means that the finding is inconsistent with the state of downstream molecule, and gray color means no prediction on the path. *The Synonyms of CYP2B6: CPB6, 2B, 2B1, 2B2, 2B3, 2B6, 2B6/7, 2B7p, 2B10, 2B19, 2B20, cyp450e, cype. § The Synonyms of CYP3A5: AI25619, cDEX, CP35, hCyp3A5 (EG1577), Cyp3A, Cyp3A1, 3A2, 3A4, m3A11, m3A16, m3A41, m3A41a, m3A41b, m3A44, m3A5, CYP450PCN3, Pcn, RL33, Rat 3A23/3A1, RatLOC100910877.

Supplementary Table S1. Primer sequences used for qPCR analysis.

Target gene	Forward (5’→3’)	Reverse (5’→3’)
Ahr	CCTTACACACAGAACTTTGC	TATGCCTGAGGACTGACC
β-Actin	TGTCCACCTTCCAGCAGATGT	AGCTCAGTAACAGTCCGCCTAGA
CAR	CTCCACTTCCATAAAAACCTGAAA	CCGAGACTGTTGTTCCATAAT
CYP1A1	AGTATTTGGTCGTGTCAGTAG	CCAGGGAAGAGTTAGGC
CYP1A2	CATAAACCAGTGGCAGGTCAA	CAGGATGGCTAAGAAGAGG
CYP2B10	CCAACCCTTGATGACCG	CAGGATTGAAACTGTCTGG
CYP3A41	CCTCTCTTTGTCATCACGG	TCCTTGAGTTTTCCACTGGT
CYP3A44	GGTGCTCCTCTACCGAT	GGGTCCGTGATGACAAG
GNMT	GTTGACGCTGGACAAAGA	AGCCTGTGCTGAGGATA
NQO1	TGCCAGCCATTCTGAAAGG	GGCAGCATATGTGTAGGCAAATC
Nrf2	GGCTGAGCAGTGAAAGG	ACCAGAAAGATGGCACC
PXR	TGGTGGCTTCCAGAAACTT	CTTCAGGGTGAGGGCAA
PPARα	CCACTACGGAGTTCACG	GGCATTCTTCCAAAGCG

Supplementary Table S2. Primer sequences used in ChIP assay.

	Forward (5'→3')	Reverse (5'→3')
CAR-XREL1	GTGCTATCCTTGAGCGT	ACCAGCCTTGTTATCTGTT
CAR-XREL2	TTTCCACGCCCTGACTT	CCAGTTTCACACTGCGTT
NRF2-XREL1	CGAGTTTGCAGCGTGGA	GAACCTTGCCCGCTTTA
NRF2-XREL2/3	AACCCCCGTTCTACGAC	CAGGCTGAAGGAGCGTG
PXR-XREL1	CTTTCCAGCCATGCGTG	GGAGAGAAATGCTCTTCGG

Supplementary methods

RNA extraction and microarray hybridization

Total liver RNA was isolated from four groups of animals, AAI- or corn oil-treated female and male WT mice, using RNeasy Mini Kits (Qiagen Inc., Valencia, CA, USA) according to the manufacturer's protocol. The RNA quality was assessed by Welgene Biotech, Co., Ltd. (Taipei, Taiwan). In each group, pooled RNA was prepared by mixing the same amount total RNA from 5 mice. The microarray hybridization was performed by Welgene Biotech using (Agilent Technologies, CA, USA). 0.2μg of total RNA was amplified by a Low Input Quick-Amp Labeling kit (Agilent Technologies, CA, USA) and labeled with Cy3 (CyDye, Agilent Technologies, USA) during the in vitro transcription process. 0.6μg of Cy3-labled cRNA was fragmented to an average size of about 50-100 nucleotides by incubation with fragmentation buffer at 60°C for 30 minutes. Correspondingly fragmented labeled cRNA is then pooled and hybridized to SurePrint G3 Mouse GE 8x60K Microarray (Agilent SurePrint Microarray, Agilent Technologies, CA, USA) at 65°C for 17 h. After washing and drying by nitrogen gun blowing, microarrays are scanned with an Agilent microarray scanner (Agilent Technologies, USA) at 535 nm for Cy3. Scanned images are analyzed by Feature extraction10.5.1.1 software (Agilent Technologies, USA), an image analysis and normalization software is used to quantify signal and background intensity for each feature. Raw signal data was normalized by quantile normalization for differential expressed genes discovering.

Immunohistochemistry

Mouse liver and kidney tissues were fixed in 10% neutral-buffered formalin for 48 hours, dehydrated through a series of graded ethanol baths and embedded in paraffin block. For IHC and H&E staining, tissue sections were deparaffinized in xylene, rehydrated in an ethanol series, antigen-retrieved in 10mM citrate buffer (pH 6.0) (60 min, 120˚C), and endogenous peroxidase

quenched with 3% H_2O_2 (30min). Sections were blocked for non-specific binding with 2% non-fat milk for 1 hour and subsequently blocked for the background of the mouse primary antibody using the Blocking Reagent A of N-Histofine® Mousestain Kit (414321F, Nichirei Bio, Inc., Tokyo, Jaapan) for 2 hours. Sections were then incubated with the primary antibody, GNMT 14-1 (1:50) (mouse mAb, YMAC Bio Tech, Taiwan), at 4˚C for 16 hrs. After blocked with the Blocking Reagent B of N-Histofine Mousestain Kit for 20 min, sections were incubated with the secondary antibody, the simple stain Mouse Max PO (M) of N-Histofine Mousestain Kit for 20 min. Next, sections were color-developed with DAB, counterstained with hematoxylin and dehydrated in graded ethanols. Finally, the sections were defatted with xylene and coverslipped with mounting medium (Histokitt No.1025/500) for light microscopy.

Western blot analysis

Culture cells were harvested with RIPA lysis buffer (50 mM Tris (pH 7.5), 150 mM NaCl, 1% Triton X-100, 0.1% SDS, 0.5% sodium deoxycholate, supplemented with protease inhibitor cocktail (Roche, Mannheim, Germany). Samples were centrifuged for 20 min at 13,000g at 4˚C. The supernatant was collected and protein concentration was determined by Bio-Rad Protein Assay (Bio-Rad Laboratories, Inc., CA, USA). 25~30μg protein were denatured with β-mercaptoethanol at 95˚C for 10min and separated by 10 % SDS-PAGE in standard SDS running buffer at 100V and subsequently transferred onto PVDF microporous membranes (PerkinElmer; NEF1002001PK) in transfer buffer at 400 mA for 80 min using the Mini-PROTEAN® Tetra electrophoresis system (Bio-Rad Laboratories, Inc., Hercules, CA, USA). The blotted membranes were blocked with 5% non-fat milk dissolved in 0.1% TBST buffer and then probed with a primary antibody at 4˚C for 16 hrs. Membranes were then incubated with a peroxidase-conjugated goat-anti rabbit as secondary antibody. ECL kit (Immobilon Western Chemiluminescent HRP Substrate, Millipore Corp, Billerica, MA, USA) was used for visualization.
